# Dissecting the Ability of Siglecs To Antagonize Fcγ Receptors

**DOI:** 10.1021/acscentsci.3c00969

**Published:** 2024-01-17

**Authors:** Kelli A. McCord, Chao Wang, Mirjam Anhalt, Wayne W. Poon, Amanda L. Gavin, Peng Wu, Matthew S. Macauley

**Affiliations:** † Department of Chemistry, University of Alberta, 11227 Saskatchewan Drive, Edmonton, Alberta T6G 2G2, Canada; ‡ Department of Molecular Medicine, Scripps Research Institute, 10550 North Torrey Pines Road, La Jolla, California 92037, United States; § Institute for Memory Impairments and Neurological Disorders, University of California, Irvine, California 92617, United States; ∥ Department of Immunology and Microbiology, Scripps Research Institute, 10550 North Torrey Pines Road, La Jolla, California 92037, United States; ⊥ Department of Medical Microbiology and Immunology, University of Alberta, Edmonton, Alberta T6G 2E1, Canada

## Abstract

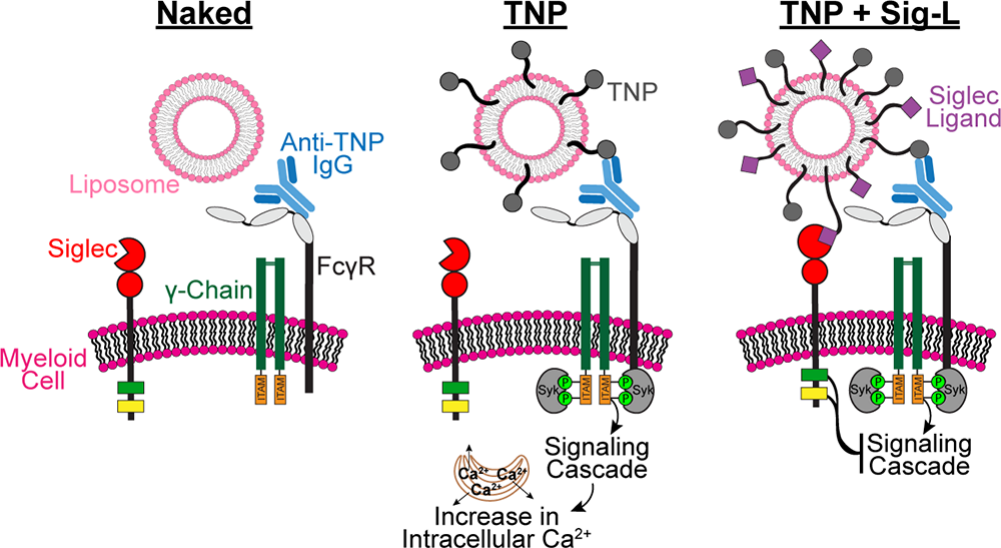

Fcγ receptors (FcγRs) play key roles in the effector function of IgG, but their inappropriate activation plays a role in several disease etiologies. Therefore, it is critical to better understand how FcγRs are regulated. Numerous studies suggest that sialic acid-binding immunoglobulin-type lectins (Siglecs), a family of immunomodulatory receptors, modulate FcγR activity; however, it is unclear of the circumstances in which Siglecs can antagonize FcγRs and which Siglecs have this ability. Using liposomes displaying selective ligands to coengage FcγRs with a specific Siglec, we explore the ability of Siglec-3, Siglec-5, Siglec-7, and Siglec-9 to antagonize signaling downstream of FcγRs. We demonstrate that Siglec-3 and Siglec-9 can fully inhibit FcγR activation in U937 cells when coengaged with FcγRs. Cells expressing Siglec mutants reveal differential roles for the immunomodulatory tyrosine-based inhibitory motif (ITIM) and immunomodulatory tyrosine-based switch motif (ITSM) in this inhibition. Imaging flow cytometry enabled visualization of SHP-1 recruitment to Siglec-3 in an ITIM-dependent manner, while SHP-2 recruitment is more ITSM-dependent. Conversely, both cytosolic motifs of Siglec-9 contribute to SHP-1/2 recruitment. Siglec-7 poorly antagonizes FcγR activation for two reasons: masking by cis ligands and differences in its ITIM and ITSM. A chimera of the Siglec-3 extracellular domains and Siglec-5 cytosolic tail strongly inhibits FcγR when coengaged, providing evidence that Siglec-5 is more like Siglec-3 and Siglec-9 in its ability to antagonize FcγRs. Additionally, Siglec-3 and Siglec-9 inhibited FcγRs when coengaged by cells displaying ligands for both the Siglec and FcγRs. These results suggest a role for Siglecs in mediating FcγR inhibition in the context of an immunological synapse, which has important relevance to the effectiveness of immunotherapies.

## Abstract 2

Liposomal nanoparticles allow coengagement of inhibitory sialic acid-binding immunoglobulin-type lectin (Siglec) receptors with Fcγ receptors to investigate regulation of immune cell activation.

## Introduction

Immune cells express cell surface Fc receptors (FcγRs) that mediate IgG antibody effector function.^1,2^ Cross-linking of FcγRs by an immune complex activates immune cells, enabling rapid response to pathogens through cellular processes such as phagocytosis, cytokine/chemokine release, production of reactive oxygen species (ROS), production of neutrophil extracellular traps (NETs), and cellular differentiation.^3−6^ The nature of the cytosolic signaling motifs on human FcγRs dictate whether they are activatory or inhibitory. Activatory FcγRs initiate immune cell signaling through the recruitment of spleen tyrosine kinase (Syk) to immunoreceptor tyrosine-based activatory motifs (ITAMs) that have been phosphorylated by Src family kinases.^7^ These ITAMs can be either located in the cytoplasmic tail of the FcγR or in a paired signaling subunit, often denoted as the γ chain.^7^ Inhibitory FcγRs balance their activatory counterparts through Src family kinase-mediated phosphorylation of immunoreceptor tyrosine-based inhibitory motifs (ITIMs) that recruit phosphatases to antagonize immune signaling.^7^ Inappropriate response of FcγRs is linked to autoimmune diseases such as systemic lupus erythematosus,^8^ rheumatoid arthritis,^9^ Kawasaki disease,^10^ and inflammatory bowel disease.^11^

Other cell surface inhibitory receptors of innate immune cells contribute to FcγR regulation. For example, ITIM-containing sialic acid-binding immunoglobulin-type lectins (Siglecs) are a family of immunomodulatory receptors that recognize sialylated glycan ligands. These ligands encompass sialic acid conjugated to a wide range of biomolecules (glycoconjugates) found throughout the body, including lipids, proteins, and other glycans. Binding of Siglecs to their sialoglycan ligands regulates the spatial proximity of Siglecs to activatory receptors and thereby modulates their ability to antagonize immune cell signaling. As sialic acid is densely displayed on all vertebrate cells, recognition of glycans by Siglecs is considered a form of “self” recognition in preventing immune activation toward normal, healthy cells.^12,13^

Immune cells of the myeloid lineage express numerous Siglec family members. While there is substantial evidence that these Siglecs are capable of regulating myeloid cell activation,^14−24^ there is still much to be learned about the activatory receptors they regulate and the physiological circumstances under which this occurs. Many inhibitory Siglecs contain a cytoplasmic ITIM (consensus sequence: (V/I/L/S)-X-Y-X-X-(L/V); X is any amino acid) and an immunoreceptor tyrosine-based switch motif (ITSM) (consensus sequence: T-X-Y-X-X-(V/I)) on their cytoplasmic tail.^25^ Phosphorylation of these motifs can recruit phosphatases and cause consequent inhibition of their coreceptors.^12,26^ There has been significant efforts aimed at understanding the ability of Siglecs to regulate FcγRs.^27−30^ While there is evidence that Siglecs can inhibit FcγRs, a systematic investigation into this ability has never been conducted.

Most tumors exhibit increased expression of sialylated ligands on their surface,^31−34^ and evidence supports Siglec–sialic acid interactions dampening or skewing antitumor immunity.^33−35^ Blocking Siglec–ligand interactions, therefore, can increase antitumor immunity.^24,27,35−39^ Antagonizing either Siglec-7 or -9 with antibodies in transgenic mice reduces tumor burden.^27^ Similarly, blockade or removal of murine Siglec-9 or Siglec-E, in microglia, increases phagocytosis of tumor cells and promotes antitumor immune responses.^35,37,39^ These findings have led Siglecs to be described as an immune checkpoint; however, the exact mechanism(s) behind this checkpoint remains to be elucidated. It was recently proposed that Siglecs expressed on myeloid cells bind to sialic acid-containing ligands on tumor cells and limit the effectiveness of tumor-targeting antibodies by antagonizing FcγRs.^27^ Moreover, antibody-mediated neutrophil cytotoxicity against tumor cells can be improved by either removing sialic acid on the surface of tumor cells or using a Siglec-9 blocking antibody.^40^ Therefore, previous evidence of Siglecs limiting the efficacy of immune checkpoints inhibitors may be the result of Siglec-mediated FcγR inhibition, leading us to question whether Siglecs can directly inhibit FcγR-mediated activation of cells. While there is evidence that Siglecs can antagonize FcγR activation, a systematic investigation into this ability has never been conducted.

**Figure 1:**
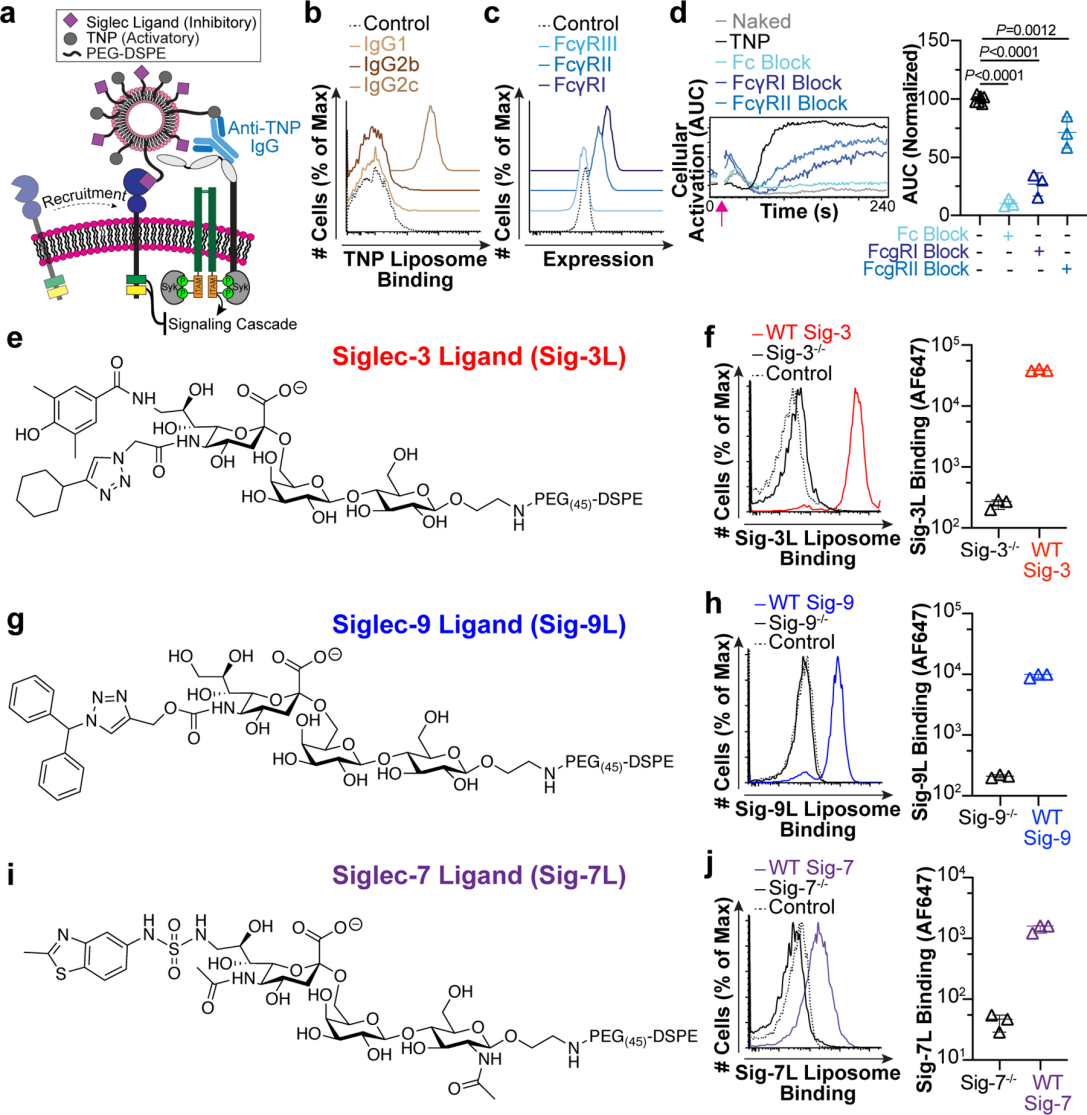
Optimizing the engagement of FcγRs and Siglecs through displaying specific ligands on liposomes. (a) Schematic of a liposomal nanoparticle platform to coengage Siglecs with FcγRs. (b) Flow cytometry histograms depicting the binding of fluorescent TNP-liposomes to cells preincubated with anti-TNP-IgG1, anti-TNP-IgG2c, or anti-TNP-IgG2b. (c) FcγR expression on U937 cells by flow cytometry. (d) Cellular activation induced by TNP-liposomes as determined by a calcium flux assay where cells were given Fc block, FcγRI block, or FcγRII block. TNP-liposomes were administered 10 s after acquisition (pink arrow). Naked liposomes were administered as a negative control. Activation was quantified by measuring the area under the calcium flux curve (area under the curve; AUC). The *P* values for three technical replicates were calculated using an unpaired Student’s *t* test, and the error bars are plotted as median with 95% confidence interval (CI). (e) Structure of selective Siglec-3 ligand (Sig-3L). (f) Flow cytometry data showing fluorescent liposomes bearing Sig-3L to U937 cells expressing Siglec-3 (WT Sig-3) and with Siglec-3 genetically removed (Sig-3^–/–^). (g, h) Chemical structure of Siglec-9 ligand (Sig-9L; g) and its ability to mediate binding of fluorescent liposomes to U937 cells with and without Siglec-9 (h). (i) New Siglec-7 ligand (Sig-7L) structure. (j) Histograms and plotted flow cytometry data of fluorescent liposomes displaying Sig-7L binding to U937 cells with and without Siglec-7. Scatter dot plots in f, h, and j, include three technical replicates, and the error bars are presented as median with 95% CI.

Motivated to develop an approach for investigating the ability of Siglecs to regulate FcγRs, we leveraged a liposomal nanoparticle platform^41^ to coengage individual Siglecs with FcγRs using highly selective glycan ligands (Figure 1A).^42−44^ We demonstrate that Siglecs-3 and -9 can fully inhibit FcγR activation on myeloid cells. The function of either the ITIM or ITSM to inhibit FcγR was dissected through their ability to recruit Src homology phosphatases. We also show that Siglec-7 poorly antagonizes FcγR activation, while a chimera between Siglec-3 and Siglec-5 demonstrates that the intracellular tail of Siglec-5 is competent at inhibiting FcγR. Furthermore, we demonstrate that Siglecs-3 and -9 are capable of inhibiting FcγR within an immunological synapse established between two cells. Overall, our results demonstrate that Siglecs-3 and -9, as well as the cytosolic tail of Siglec-5, can potently antagonize FcγRs, which may be a mechanism that limits the effectiveness of immunotherapies.

## Results and Discussion

### Liposomes To Engage FcγRs

Human peripheral blood neutrophils and monocytes express Siglecs-3, -5, -7, and -9 (Figure S1A,B). To study the role of Siglecs in regulating FcγRs, the human monocytic cell line U937 was used, which naturally expresses Siglecs-3, -5, and -7 (Figure S1C). U937 cells were chosen because (i) they have previously been used to study the function of both FcγRs and Siglecs,^28,29^ (ii) their FcγRs are relatively free due to reduced levels of IgG in culture media, unlike primary monocytes whose FcγRs are mostly saturated by IgG,^45−47^ and (iii) they can be genetically manipulated to investigate mechanism. U937 cell lines with or without each Siglec were developed by first disrupting the expression of each Siglec by CRISPR/Cas9, followed by reintroduction of each Siglec through lentiviral transduction or an empty vector control under the EF1α promoter (Figure S2). These cell lines were used to test the ability of each Siglec to antagonize FcγRs by using liposomes codisplaying a high affinity, Siglec-specific ligand along with a ligand to engage FcγRs.

Prior to codisplaying the two ligands, we individually optimized the engagement of Siglecs and FcγRs. To engage FcγRs, we developed a set of mouse monoclonal anti-trinitrophenyl (TNP) IgG antibodies of the IgG1, IgG2c, and IgG2b isotypes (Figure S3). To engage these antibodies, TNP was conjugated to 1,2-distearyol-*sn*-glycero-3-phosphoethanolamine-poly(ethylene glycol) (PEG–DSPE) (Figure S4), allowing for multivalent presentation of TNP from the liposomes. U937 cells were preincubated with anti-TNP-IgG1, anti-TNP-IgG2c, or anti-TNP-IgG2b, washed, probed with fluorescent TNP-liposomes, and measured by flow cytometry. Preincubation with anti-TNP-IgG2c resulted in significant liposome binding, while preincubation with anti-TNP-IgG1 or anti-TNP-IgG2b resulted in little to no binding (Figure 1B). This is in line with mouse IgG2a/c having the highest affinity for human FcγRI,^48^ and FcγRI being highly expressed on U937 cells (Figure 1C). The amount of TNP–PEG–DSPE on the liposome was titrated, and significant binding was achieved with as little as 0.1 mol % of TNP–PEG–DSPE (Figure S5A,B). Thus, moving forward we used 0.1 mol % of TNP–PEG–DSPE in liposomes to stimulate FcγRs. TNP-liposome binding to cells incubated with anti-TNP-IgG2c was decreased upon prior treatment of cells with Fc-blocking antibodies, indicating that the binding was FcγR dependent (Figure S5C,D). Consistent with a previous report,^2^ U937 cells express FcγRI at a high level, with slightly less FcγRII, and minimal FcγRIII (Figure 1C), which is similar to their expression pattern on human peripheral blood monocytes (Figure S6A,B).

To test the ability of Siglecs to regulate cellular activation through FcγRs, a calcium flux assay was employed. This involved monitoring calcium flux in the cytoplasm using cells loaded with the ratiometric fluorophore, Indo-1-acetoxymethyl ester (Indo-1-Am). U937 cells incubated with anti-TNP IgG2c showed a robust calcium flux upon stimulation with TNP liposomes (Figure 1d). Activation was fully abrogated by preincubating cells with Fc-blocking antibodies, while selectively blocking FcγRI inhibited calcium flux by 90% and selectively blocking FcγRII inhibited calcium flux by 30% (Figure 1d). This suggests that FcγRI plays the dominant role in the response of U937 cells to cross-linking of immobilized anti-TNP-IgG2c. The dominance of FcγRI in this assay is consistent with FcγRI (i) being expressed at high levels on U937 cells (Figure 1c), and (ii) having a higher affinity toward monomeric antibodies and, in particular, the IgG2a/c isotype, compared to FcγRII,^6,8^ leading to better liposome binding (Figure 1b).

### Liposomes To Engage Siglecs

We optimized the binding of liposomes to individual Siglecs using high affinity and selective Siglec ligands composed of sialosides bearing a chemically modified sialic acid with hydrophobic groups appended from the fifth and/or the ninth carbon. Siglec-3 and -9 ligands were previously developed.^49−51^ These ligands were linked to PEGylated lipids and incorporated into liposomes containing a fluorophore (Alexa Fluor 647; AF647), as described previously,^52^ to assess fluorescent liposome binding to cells by flow cytometry. Liposomes displaying AF647–PEG–DSPE and the Siglec-3 ligand (Sig-3L; Figure 1e) readily bound WT Siglec-3 cells but not Siglec-3^–/–^ cells (Figure 1f). Similarly, liposomes displaying AF647–PEG–DSPE and a Siglec-9 ligand (Sig-9L; Figure 1g) bound to Siglec-9 expressing cells but not Siglec-9^–/–^ cells (Figure 1h). A high affinity Siglec-7 ligand was previously described,^53^ but in our hands this ligand was not selective for only Siglec-7. Therefore, we developed a Siglec-7 ligand (Sig-7L; Figure 1i) that binds to Siglec-7-expressing cells but not Siglec-7^–/–^ cells (Figure 1j). Efforts to discover a ligand for targeting Siglec-5 have, thus far, been unsuccessful, and an alternative approach was used later to examine the inhibitory ability of Siglec-5.

### Testing the Intrinsic Ability of Siglecs To Inhibit FcγRs

**Figure 2:**
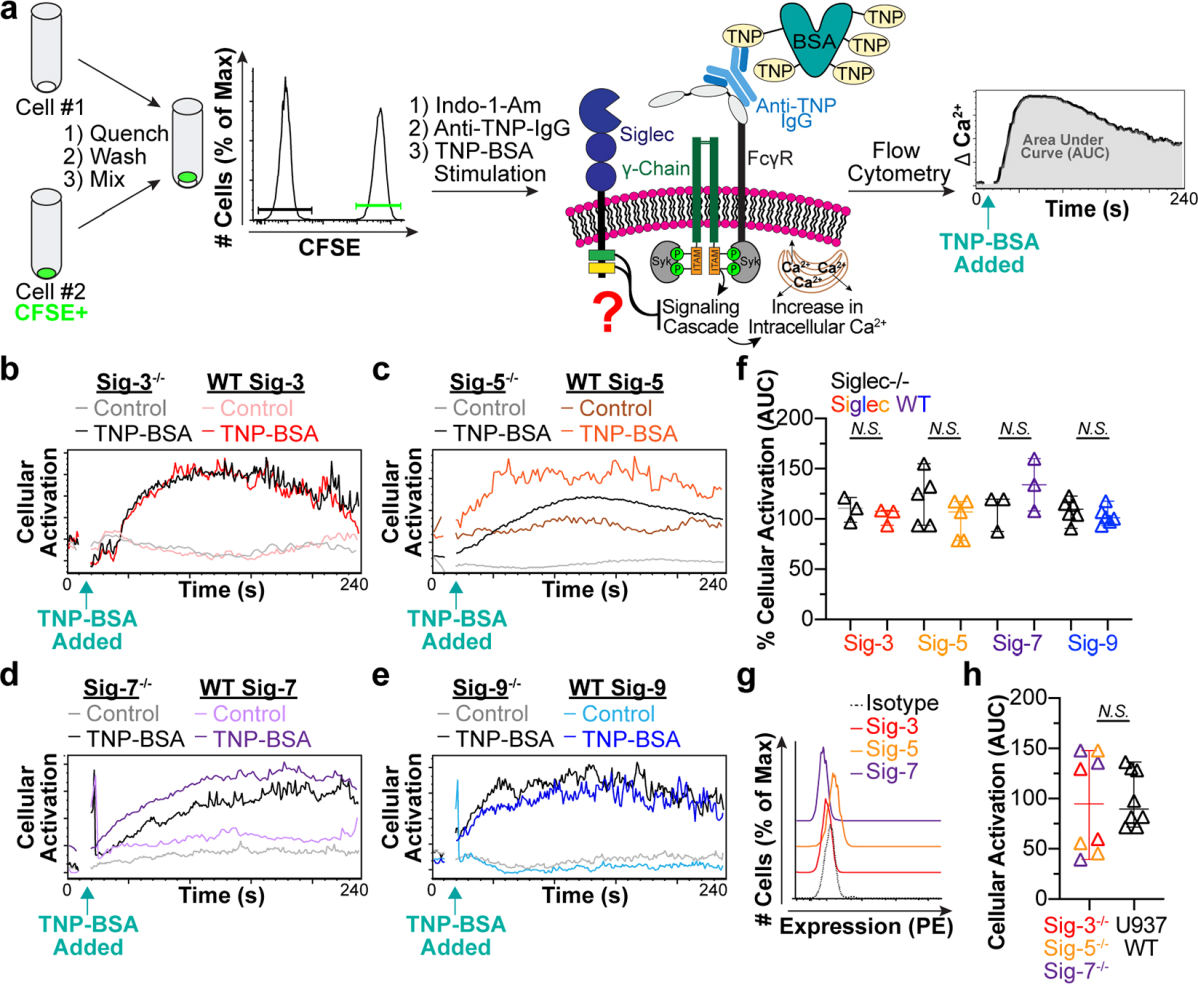
Siglecs do not intrinsically inhibit FcγR activation. (a) Schematic of an internally controlled assay to examine calcium flux where one cell line is labeled with CFSE dye and mixed with another unstained cell line. The mixture of cells is loaded with Indo-1-Am followed by incubation with anti-TNP-IgG2c antibody. After 10 s to establish a baseline, TNP-BSA is added to the cells, and the change in calcium is monitored over 240 s. (b–e) Example calcium flux of Siglec^–/–^ and WT Siglec cells administered TNP-BSA versus media (control) over 240 s. (f) AUC calculated from calcium flux data. The plotted values were adjusted by subtracting the AUC of the Naked liposome and were further normalized to the level of TNP liposome activation. Each data point represents one technical replicate performed. *P* values were calculated using an unpaired Student’s *t* test with three, five, three, or six technical replicates for Sig-3, -5, -7, and -9, respectively. (g) Siglec expression on CRISPR triple knockout (Siglec-3^–/–^ Siglec-5^–/–^ Siglec-7^–/–^) U937 cells. (h) The amount of cellular activation (AUC) from calcium flux of Siglec-3^–/–^ Siglec-5^–/–^ Siglec-7^–/–^ cells run in parallel with U937 WT cells with Naked liposome AUC subtracted. The P value was calculated for eight technical replicates using an unpaired Student’s *t* test. Error bars in f and h represent median with 95% CI.

Before testing the impact of coengaging a Siglec with FcγRs, we tested their intrinsic ability to do so without forced coengagement. Specifically, we tested activation of FcγRs in cells with and without an individual Siglec. An internally controlled assay was developed in which one set of cells was labeled with carboxyfluorescein succinimidyl ester (CFSE) dye. These stained cells could then be mixed with a different set of unstained cells to examine the activation of two cell types under the same conditions, at the same time (Figure 2a). Mixed cells were loaded with Indo-1-Am and incubated with anti-TNP-IgG2c antibodies. Trinitrophenyl-bovine serum albumin (TNP-BSA) was used to stimulate the cell mixture, and calcium flux was monitored by flow cytometry. Because highly multivalent antigens have been seen to exclude inhibitory receptors,^54^ TNP-BSA was initially chosen to assess intrinsic Siglec inhibition, as it is less multivalent than liposomes. The results demonstrated that the presence of an individual Siglec does not impact the cellular activation (Figure 2b–f). To ensure that there was no redundancy between the three Siglecs naturally expressed on U937 cells, we created CRISPR/Cas9 gene-edited triple-knockout U937 cells (deficient in Siglecs-3, -5, and -7) (Figure 2g). Triple knockout cells also showed comparable levels of activation through FcγR compared to WT cells (Figure 2h). Similar results were also obtained with TNP liposomes (Figure S7). These results indicate that Siglec expression alone does not impact cellular activation through the FcγR.

### Coengagement of Siglecs-3 or -9 with FcγRs Inhibits Cellular Activation

**Figure 3:**
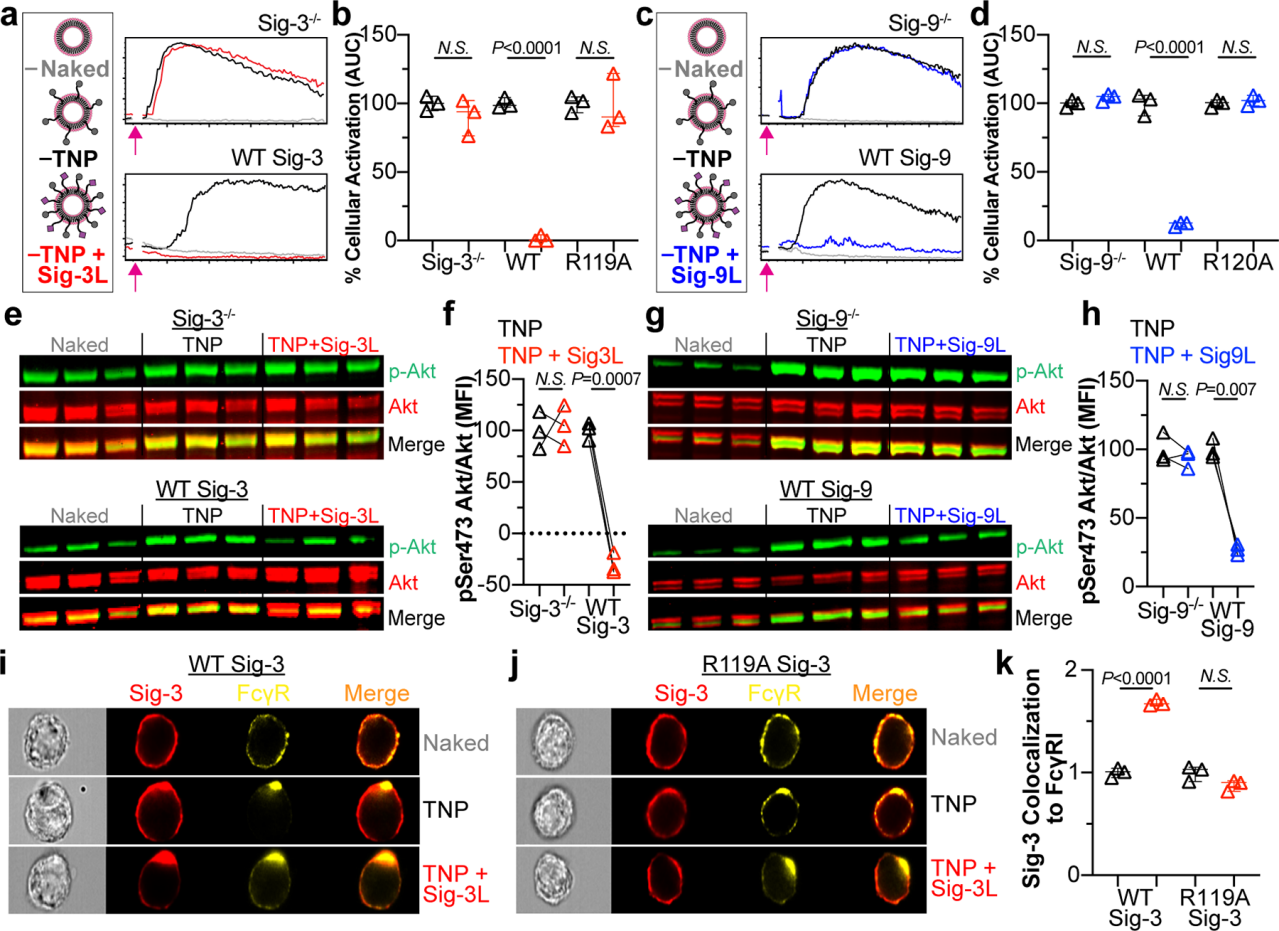
Siglecs-3 and -9 can fully inhibit FcγR activation. (a) Representative calcium flux in Siglec-3^–/–^ or WT Siglec-3 cells stimulated with the indicated liposomes. The pink arrows represent the addition of liposomes at 10 s. (b) The amount of cellular activation after administration of indicated liposomes to Siglec-3^–/–^, WT Siglec-3, or R119A Siglec-3 cells is quantified by the AUC with the Naked liposome AUC subtracted and normalized to the amount of activation from TNP liposomes. Three technical replicates are shown. (c) Representative calcium flux in Siglec-9^–/–^ or WT Siglec-9 cells after being given the denoted liposomes at 10 s. (d) Quantification of cellular activation by calcium flux for Siglec-9^–/–^, WT Siglec-9, or R120A Siglec-9 cells after administration of the indicated liposomes. Three technical replicates are shown. (e, f, g, h) Western blot of Akt and p-Akt (pSer473) in Siglec-3^–/–^ and WT Siglec-3 cells (e) or Siglec-9^–/–^ and WT Siglec-9 cells (g) administered Naked liposomes, TNP liposomes, or TNP and 1% Sig-L liposomes. The ratio of pSer473 Akt fluorescent signal over Akt fluorescent signal was quantified using ImageStudio Lite Software. (f,h). Three technical replicates are plotted. Statistical analysis was performed using a paired Student’s *t* test. (i, j) Representative images of imaging flow cytometry data investigating Siglec-3 and FcγRI colocalization after administration of indicated liposomes to WT Siglec-3 cells (i) or R119A Siglec-3 cells (j). (k) Quantification of Siglec-3 and FcγRI colocalization using IDEAS Software. Each data point represents a technical replicate and is an average of 8000 cells. All P values were calculated using an unpaired Student’s *t* test with three technical replicates. Data plots in b, d, and k are presented as median with 95% CI.

We next examined the impact of coengaging Siglecs with FcγRs. Specifically, we measured cellular activation of U937 cells incubated with anti-TNP IgG2c using three types of liposomes: liposomes without ligands (Naked), liposomes that display TNP alone (TNP), and liposomes that display both TNP and a Siglec ligand (TNP + Sig-L) (Figure 3A; Figure S8A). Naked liposomes (not expected to activate cells) were used to set the baseline for cellular activation. To complement the set of Siglec^–/–^ cells reintroduced with an empty vector control or WT Siglec, we generated cells expressing Siglecs with their critical arginine residue, which is required for binding, mutated to alanine (Figure S9). Consequently, the cells with mutated Siglec are unable to interact with their corresponding sialoside ligand.

U937 cells expressing WT Siglec-3 showed robust activation when stimulated with TNP liposomes. However, they showed no activation to TNP + Sig-3L liposomes (Figure 3a,b; Figure S8B). In Siglec-3^–/–^ and R119A Siglec-3 cells, calcium flux with TNP + Sig-3L liposomes compared to TNP liposomes did not differ, demonstrating that the presence of Sig-3L on the liposome did not alter the ability of TNP to activate the cells. Importantly, separate liposomes displaying only Sig-3L or TNP administered simultaneously did not result in decreased cellular activation as measured by calcium flux (Figure S10A,B), and WT U937 cells expressing endogenous Siglec-3 also exhibited decreased FcγR activation when administered TNP + Sig-3L liposomes (Figure S10C,D). These observations indicate that the two receptors must be coengaged by ligands on the *same* liposome and strongly suggest that Siglec-3 and FcγRs must be brought into proximity for Siglec-3 to exert its inhibitory effect. An analogous set of experiments revealed that Siglec-9 is also capable of potently inhibiting FcγRs (Figure 3c,d, Figure S8C). Liposomes precomplexed with the anti-TNP-IgG2c also bound to U937 cells (Figure S11A,B). Excess anti-TNP-IgG2c outcompeted binding of precomplexed anti-TNP-IgG2c on liposomes. Optimized precomplexed conditions led to stimulation of U937 cells and anti-TNP-IgG2c precomplexed with liposomes containing Sig-3L did not activate U937 cells (Figure S11C,D).

As a second method to monitor cellular activation through the FcγR, we examined the phosphorylation of downstream activation proteins, protein kinase B (Akt) and extracellular signal-regulated kinase (Erk), by Western blotting following liposome stimulation. Akt and Erk phosphorylation was increased in response to TNP liposome activation when compared to Naked liposomes. This signaling was abrogated in cells expressing WT Siglec-3, but not Siglec-3^–/–^ cells, stimulated with TNP + Sig-3L liposomes (Figure 3e,f, Figure S12A,B**)**. Similar results were observed for Siglec-9 when using TNP + Sig-9L liposomes (Figure 3g,h, Figure S12C,D). Together with the calcium flux data, these results demonstrate Siglec-3 or -9 -dependent inhibition of FcγRs when the receptors are coengaged.

Previous evidence of antibody-mediated cross-linking of Siglecs with FcRs has demonstrated the inhibitory ability of Siglecs.^17,20−22,30^ This was accomplished by administering anti-FcγRI and anti-Siglec-3 antibodies to U937 cells followed by a secondary antibody to coligate the two receptors.^28,29^ A similar approach was used to examine Siglec-5, -7, -8, and -9 inhibition of FcεRs on mast cells, basophils, or rat basophilic leukemia cells.^17,20−23^ This approach makes it difficult to rule out the possibility that the anti-Siglec antibody hinders the extent of FcR cross-linking by the secondary, which may not necessarily represent true Siglec-dependent inhibition. The liposome platform helps overcome this limitation and has previously been used to dissect the ability of Siglec-3 and -8 to antagonize FcεRI on mast cells^43,55^ as well CD22 and Siglec-G on B cells.^42,56^

### Visualization of Coengagement of Siglecs with FcγRI

To examine if the liposomes displaying TNP and Sig-3L were bringing FcγRI and Siglec-3 together, we used imaging flow cytometry to visualize Siglec-3 and FcγRI 2 min after stimulation with liposomes. In WT Siglec-3 cells, Siglec-3 and FcγRI were uniformly distributed around the cell surface when administered Naked liposomes (Figure 3i). When stimulated with TNP liposomes, FcγRI clustered together, but Siglec-3 remained dispersed. Administration of TNP + Sig-3L liposomes led to overlap of the two fluorescent signals, strongly suggesting that the two receptors were clustered together. Importantly, cells expressing R119A Siglec-3 did not result in colocalization of Siglec-3 with FcγRI when given TNP + Sig-3L liposomes (Figure 3j,k). Furthermore, using liposomes displaying TNP, Sig-3L, and AF647, we were further able to demonstrate the colocalization of the FcγRI, Siglec-3, and liposomal nanoparticles (Figure S13). These results support the hypothesis that liposomes codisplaying a Siglec ligand and antigen bring together the Siglec with FcγRI, allowing the Siglec to inhibit receptor driven signals.

### Both the ITIM and ITSM of Siglecs-3 and -9 Contribute to Inhibition

**Figure 4:**
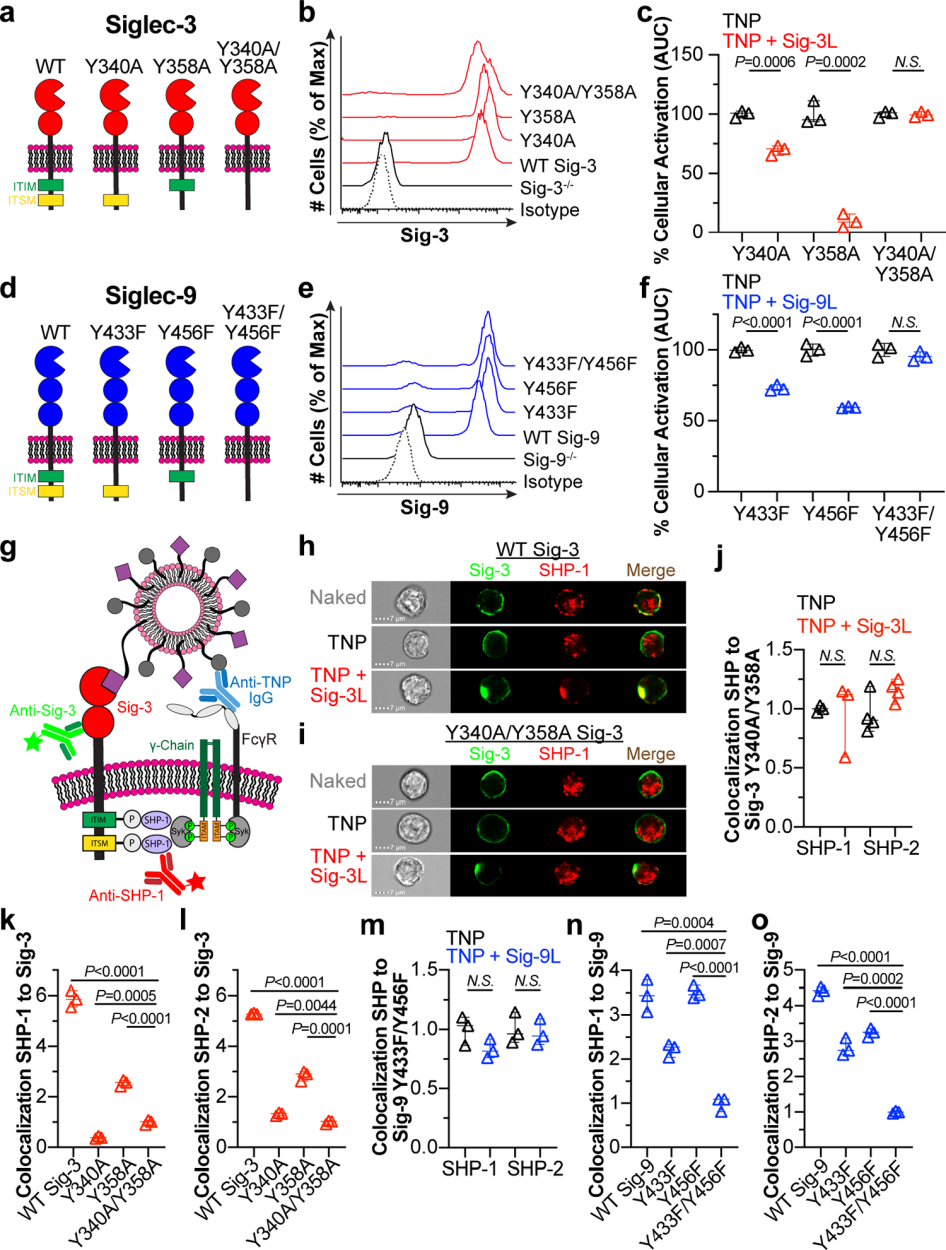
Evaluating the contribution of the ITIM and ITSM in Siglecs-3 and -9 mediated inhibition of FcγRs. (a) Schematic of Siglec-3 cytosolic motif mutants. (b) Siglec-3 staining of U937 cells virally transduced with cytosolic motif mutants. (c) Quantification of cellular activation by calcium flux (AUC) after administration of the denoted liposomes in cells expressing Y340A, Y358A, or Y340A/Y358A Siglec-3. The Naked liposome AUC is subtracted, and the resulting AUC is normalized to the amount of activation from TNP liposomes. Three technical replicates are plotted. (d) Schematic of Siglec cytosolic motif mutants. (e) Flow cytometry staining of Siglec-9 in cytosolic motif mutants. (f) Calcium flux AUC of the cytosolic mutant cells expressing Y433F, Y456F, or Y433F/Y456F Siglec-9 after administration of the indicated liposomes. The plotted values are three technical replicates, have the AUC of the Naked liposome subtracted, and are normalized to the amount of activation from TNP liposomes. (g) Illustration of the imaging flow cytometry methodology. (h, i) Representative imaging flow cytometer images of Siglec-3 colocalization to SHP-1 in U937 cells expressing WT Siglec-3 (h) or Y340/Y358A Siglec-3 (i) when administered the indicated liposomes. (j) Quantification of the amount of colocalization of SHP-1 and SHP-2 to Siglec-3 in WT Siglec-3 and Y340A/Y358A Siglec-3 cells. (k, l) Colocalization of Siglec-3 to SHP-1 (k) and SHP-2 (l) in U937 cells expressing Y340A/Y358A, WT Siglec-3, Y340A, or Y358A when administered TNP + Sig-3L liposomes. All values are normalized to Y340A/Y358A Siglec-3. (m) Colocalization of SHP-1 and SHP-2 to Siglec-9 in WT Siglec-9 and Y433F/Y456F Siglec-9 cells. (n, o) Siglec-9 colocalization to SHP-1 (n) and SHP-2 (o) in U937 cells expressing Y433F/Y456F, WT Siglec-9, Y433F, and Y456F after incubation with TNP + Sig-9L liposomes. Values are normalized to Y433F/Y456F Siglec-9. IDEAS Software was used for quantification of colocalization data in j–o in which each data point represents a technical replicate and average of 8000 cells. Data plots c, f, i–o have error bars presented as median with 95% CI, and statistical analysis was performed on three technical replicates for each condition using unpaired Student’s *t* tests.

To investigate the contributions of the ITIM and ITSM to inhibition of FcγRs by Siglecs-3 and -9, we introduced point mutants of these Siglecs back into the Siglec^–/–^ U937 cells. For Siglec-3, the ITIM mutant (Y340A), ITSM mutant (Y358A), or ITIM and ITSM double mutant (Y340A/Y358A) was used (Figure 4a). Flow cytometry staining of each of these mutant cell lines revealed similar cell surface expression levels (Figure 4b). Using calcium flux to measure cellular activation through the FcγR, we observed that Y340A Siglec-3 lost a significant amount of its ability to repress calcium flux in response to the TNP + Sig-3L liposomes relative to TNP liposomes, but approximately 30% inhibition was still observed (Figure 4c). Y358A Siglec-3 was capable of inhibiting calcium flux by 90% while coengaging Y340A/Y358A Siglec-3 with FcγRs did not result in inhibition. These results indicate that both motifs contribute to the ability of Siglec-3 to inhibit FcγRs, with the ITIM playing a more significant role.

For Siglec-9, the ITIM mutant (Y433F), ITSM mutant (Y456F), or ITIM and ITSM double mutant (Y433F/Y456F) was used (Figure 4d). Viral transduction of these constructs into Siglec deficient U937 cells resulted in expression of these mutants at relatively similar levels to WT Siglec-9 expression (Figure 4e). Y433F and Y456F inhibited activation by 30 and 40%, respectively, when stimulated with TNP + Sig-9L liposomes relative to TNP liposomes, while Y433F/Y456F showed no inhibition (Figure 4f). These results indicate that both the ITIM and ITSM contribute to the ability of Siglec-9 to inhibit FcγRs.

### Siglecs-3 and -9 Recruit SHP-1 and SHP-2 to Their Cytosolic Motifs

Phosphorylation of the ITIM on Siglecs recruits phosphatases such as SHP-1, SHP-2, and SH2 domain-containing inositol polyphosphate-5-phosphatases (SHIPs) to dampen immune cell signaling.^26,38,57,58^ Nevertheless, the contributions of the ITSM has been less thoroughly investigated. For Siglec-3, roles for its ITIM in mediating inhibition through SHP-1^28,57^ and SHP-2^57^ are known. There is also some evidence pointing toward both the ITIM and ITSM of Siglec-3 being phosphorylated after treatment with a protein tyrosine phosphatase inhibitor, pervanadate,^59^ and our calcium flux data revealed a dominant role for Siglec-3 ITIM in inhibiting FcγRs, with a minor role for the ITSM. To further probe the role of the ITIM and ITSM, we employed an imaging flow cytometry assay to visualize colocalization of Siglec-3 with SHP-1 and SHP-2 (Figure 4g). When U937 cells expressing WT Siglec-3 were stimulated with Naked liposomes, SHP-1 staining was distributed inside the cell (Figure 4h). This appearance was not significantly altered when cells were stimulated with TNP liposomes. However, when the cells were stimulated with TNP + Sig-3L liposomes, Siglec-3 and SHP-1 overlapped in a punctate signal near the cell surface (Figure 4h), strongly suggesting that SHP-1 was being recruited to Siglec-3 cytoplasmic tail. The Y340A/Y458A double mutant of Siglec-3 did not show colocalization of Siglec-3 with SHP-1 as the SHP-1 signal remained distributed inside of the cell when the Siglec was engaged by the liposome (Figure 4i,j). Similar results were obtained for SHP-2 (Figure 4j; Figure S14A,B). In contrast, no evidence was found for the recruitment of SHIP-1 or SHIP-2 to Siglec-3 (Figure S15A).

Having established that the ITIM and ITSM are necessary for SHP-1/2 recruitment upon coligation of Siglec-3 and FcγRs, we examined the contribution of each motif. To do so, we normalized the amount of Siglec-3 and SHP-1/2 colocalization in mutant cell lines when given TNP + Sig-3L liposomes to the amount of colocalization in Y340A/Y358A cells. Data were normalized to the double mutant because it did not recruit SHP-1 or SHP-2 and, therefore, provided a baseline. Compared to the amount of colocalization of SHP-1 with WT Siglec-3, the Y340A mutant showed no colocalization with SHP-1, while the Y358A had some colocalization, albeit less than WT Siglec-3 (Figure 4k). For SHP-2 colocalization with Siglec-3, similar results were found with WT Siglec-3 and Y358A Siglec-3. Additionally, we observed a small yet significant amount of SHP-2 recruitment to Y340A Siglec-3 (Figure 4l). These results are consistent with the calcium flux inhibition data, indicating that the ITIM plays a more dominant role in inhibition, as the ITIM alone (Y358A) significantly recruits more SHP-1 and SHP-2 than the ITSM (Figure 4k,l).

We also investigated colocalization of SHP-1/-2 with Siglec-9 and found that both phosphatases displayed enhanced localization with WT Siglec-9 after stimulation of TNP + Sig-9L liposomes, but not the Y433F/Y456F double mutant (Figure 4m; Figure S14C–F). Again, the amount of Siglec-9 and SHP-1/2 colocalization in each cell line was normalized to the amount of colocalization in the Y433F/Y456F cells when administered TNP + Sig-9L liposomes (Figure 4n,o). Here, mutation of the ITSM (Y456F) behaved similar to that of Siglec-3 in that the mutant was still able to recruit both SHP-1 and -2. Unlike Siglec-3, however, mutation of the ITIM (Y433F) also resulted in colocalization of both SHP-1 and SHP-2 (Figure 4n,o). These data suggest that both the ITIM and ITSM of Siglec-9 participate in the recruitment of SHP-1 and SHP-2. No significant SHIP-1 or SHIP-2 colocalization to Siglec-9 was observed (Figure S15B).

Previously, biotinylated peptides containing the phosphorylated ITIM or ITSM of Siglec-3 were used to capture proteins from U937 cell lysates in which the captured proteins included SHP-1 and SHP-2.^29^ This indicated that the Siglec-3 ITIM peptide bound to both SHP-1 and SHP-2, but the ITSM peptide only bound to SHP-2.^29^ These previous data are consistent with our imaging flow cytometry findings that Siglec-3 ITIM can recruit SHP-1 and SHP-2 phosphatases for inhibition, but the ITSM primarily recruits SHP-2. Previous work examining phosphatase recruitment to Siglec-9 upon pervanadate-mediated stimulation revealed that Siglec-9 can recruit both SHP-1 and SHP-2,^20,60^ but does not recruit SHIP-1 and SHIP-2.^60^ These findings further support our imaging flow cytometry quantification demonstrating that Siglec-9 can recruit both SHP-1 and SHP-2 after liposome stimulation. It is important to note that the majority of previous work involved the use of pervanadate to induce phosphorylation,^20,28,29,57,60^ which does not represent physiological conditions for Siglec phosphorylation. Our liposome platform represents a more physiologically relevant circumstance where a Siglec and FcγRs are brought together on the cell surface.

The differential recruitment of SHP-1 and SHP-2 by Siglec-3 and Siglec-9 is interesting given that their ITIM and ITSM sequences only slightly differ from one another, with a His residue in Siglec-3 ITIM being a Gln residue in Siglec-9 ITIM and a Val residue in Siglec-3 ITSM being an Ile in Siglec-9 ITSM (Figure S16). It is possible that the His in Siglec-3 ITIM may help its inhibition ability; however, another key distinction between Siglecs-3 and -9 is the spacing between the two cytosolic motifs. For Siglec-3, they are 12 bases apart whereas they are 17 bases apart in Siglec-9. A previous report suggested that the spacing between the two tyrosine binding sites may be important for the differential binding of enzymes.^61^ An intriguing analogy is how SHP-1 and SHP-2 are differentially recruited by PD-1 and BTLA. While both proteins have an ITIM and an ITSM, PD-1 mainly uses its ITSM to recruit SHP-2, while BTLA mainly uses its ITIM to recruit SHP-1.^62^ This is very interesting as their ITSM consensus sequences are nearly identical and, moreover, the distance between their ITIM and ITSM for both proteins are also identical. This illustrates that many factors may be at play when considering the ability of a receptor to recruit SHP-1 versus SHP-2.

### Siglec-7 Does Not Fully Inhibit FcγR Activation

**Figure 5:**
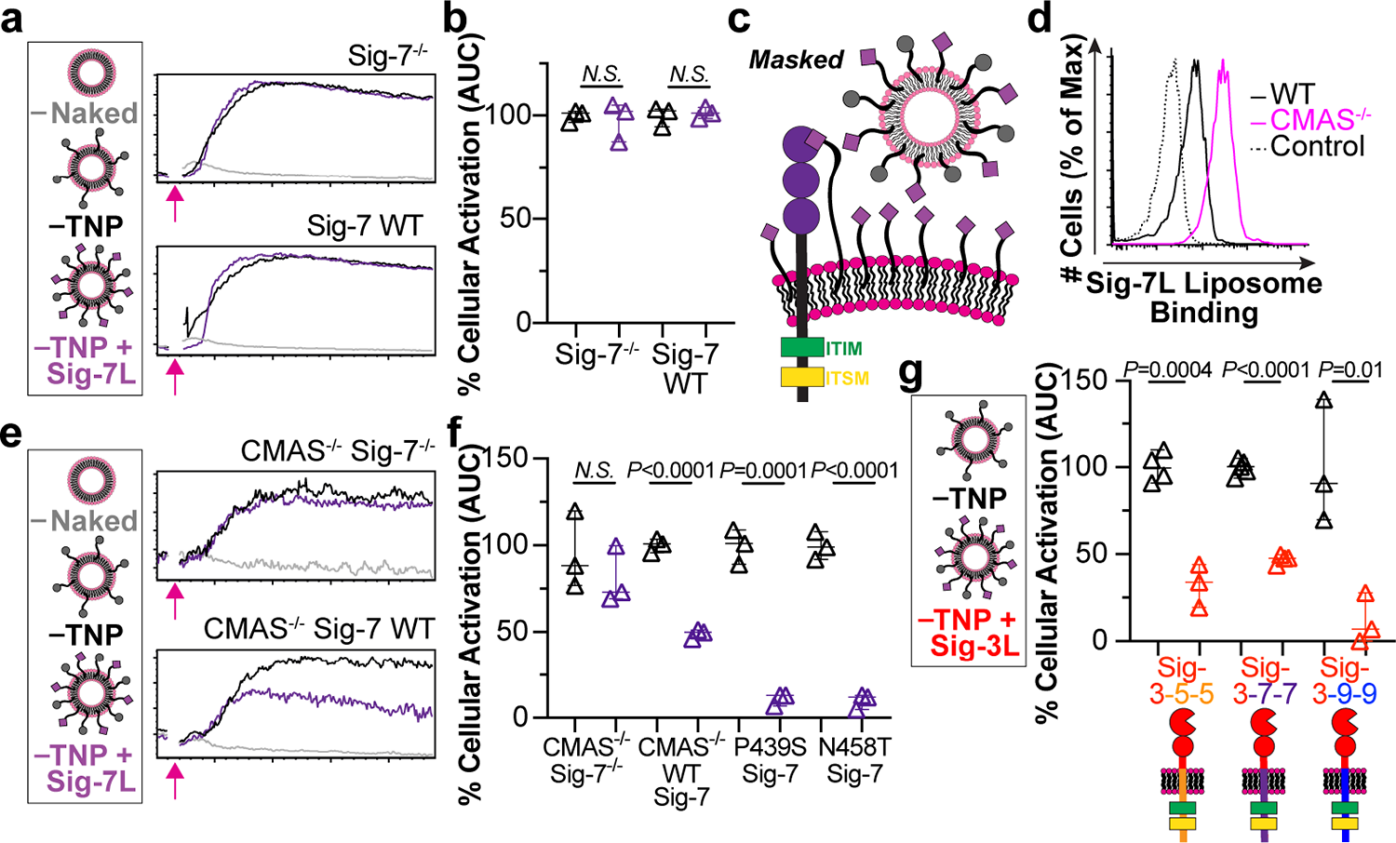
Siglec-7 does not fully inhibit FcγR activation. (a) Representative calcium flux in Siglec-7^–/–^ and WT Siglec-7 cells stimulated with the indicated liposomes as measured by a flow cytometer. The pink arrow illustrated the addition of liposomes after acquisition. (b) AUC from calcium flux plotted as a measure of cellular activation. The AUC from the background, Naked liposomes, was subtracted, and the values are normalized to activation from TNP liposomes. (c) Schematic hypothesis of sialic acid cis-binding masking liposome binding to Siglec-7. (d) Flow cytometry histograms showing Sig-7L displaying liposomes binding to cells with cytidine monophospho-*N*-acetylneuraminic acid synthetase (CMAS) genetically removed. (e) Calcium flux of CMAS^–/–^ Siglec-7^–/–^ cells compared to CMAS^–/–^ WT Siglec-7 cells when given the indicated liposomes. (f) AUC from calcium flux of CMAS^–/–^ Siglec-7^–/–^ cells virally transduced with empty vector (Siglec-7^–/–^), WT Siglec-7, P439S Siglec-7, or N458T Siglec-7 administered TNP or TNP + Sig-7L liposomes. AUC is plotted with Naked liposomes subtracted and normalized to TNP liposomes. (g) Quantified AUC from calcium flux of Sig-3–5–5, 3–7–7, and 3–9–9 chimera when given the indicated liposomes. Values have AUC from Naked liposomes subtracted and are normalized to activation when given TNP liposomes. All data points represent technical replicates and statistical analysis was performed using an unpaired Student’s *t* test. Data in plots in b, f, and g are three technical replicates presented as median with 95% CI.

Unlike Siglecs-3 and -9, WT Siglec-7 cells did not show a significant difference in cellular activation when given TNP and TNP + Sig-7L liposomes (Figure 5a,b; Figure S9C). Because Siglec-7 has been shown to be masked by cis interactions with ligands,^12,63−67^ we hypothesized that this may hinder the ability of Siglec-7 to inhibit FcγRs (Figure 5c). Strong masking of Siglec-7 was confirmed through greatly enhanced binding of Sig-7L liposome binding to cells treated with neuraminidase to remove cis ligands (Figure S17A). U937 cells lacking sialic acid, through an introduced mutation in cytidine monophospho-*N*-acetylneuraminic acid synthetase (CMAS),^49,68^ also displayed significantly increased Sig7-L liposome binding compared to WT cells (Figure 5d). We deleted Siglec-7 in the CMAS^–/–^ cells and then virally transduced them with empty lentiviral control or WT Siglec-7 (Figure S17B). CMAS^–/–^ cells overexpressing Siglec-7 bound Sig-7L liposomes very well (Figure S17C). Cellular activation studies through FcγRs were performed using the CMAS^–/–^ cells, which revealed that coengaging Siglec-7 inhibited activation by 50% (Figure 5e,f). This implies that Siglec-7 is less effective at inhibiting FcγRI when compared to Siglec-3 and -9.

Sequence alignment of the ITIM and ITSM sequences of Siglecs-3, -5, -7, and -9 reveals two unique features for Siglec-7 within the motifs: a proline in the ITIM and an asparagine in the ITSM of Siglec-7 as opposed to serine and threonine, respectively (Figure S16). In fact, because Siglec-7 does not follow an ITSM consensus sequence, T-X-Y-X-X-(V/I), its intracellular motif is referred to as an ITIM-like motif having a consensus sequence (D/E)-X-Y-X-(EV/IK/R).^25,69^ To make the Siglec-7 motifs better align with Siglec-3, -5, and -9, we created P439S and N458T mutants and transduced them into CMAS^–/–^ Sig-7^–/–^ cells to yield cells with similar Siglec-7 expression levels and Sig-7L liposome binding (Figure S18A–D). Using our calcium flux assay, we compared their ability to inhibit FcγRs in response to TNP + Sig-7L liposomes. We observed that, compared to WT Siglec-7, both P439S and N458T mutants had enhanced ability to inhibit FcγRs (Figure 5f).

Previously, Siglec-7 was shown to recruit SHP-1 and SHP-2 to a lesser extent than Siglec-9 upon pervanadate stimulation and mutation of P439 or N458 increased SHP-1/2 recruitment to Siglec-7.^20,60,70^ This is consistent with our results demonstrating that is Siglec-7 is not a strong antagonist of FcγRs compared to Siglecs-3 and -9. It is interesting to speculate that Siglec-7 may be more specialized at regulating other activatory receptors in other cell types. Notably, there is evidence demonstrating that Siglec-7 can inhibit a different type of FcR, FcεRI, on mast cells and basophils^21^ as well as rat basophilic leukemia cells.^20^ Differences in the inhibitory ability of Siglec-7 toward FcRs may stem from differences in cis ligands for Siglec-7 present on different cell types or intrinsic differences between the types of FcR. A previous study examining Siglecs-7 and -9 on natural killer (NK) cells also demonstrated that engagement of either Siglec via an antibody resulted in inhibition of NK cell activation.^65^ Cancer cells can take advantage of this phenomenon and up-regulate their Siglec ligands to inhibit NK cell activation and escape destruction. As NK cells primarily express FcγRIII, which can also be activated upon cross-linking,^71,72^ it will be of interest to investigate the ability of Siglec-7 and -9 to more broadly inhibit FcγRIII on NK cells.

### Creating Chimeric Siglecs To Investigate the Ability of Siglec-5 To Inhibit FcγRs

Without a ligand to engage Siglec-5, we developed three chimeric proteins consisting of the extracellular domains of Siglec-3 with the transmembrane domain and cytosolic tails of Siglec-5 (3–5–5), Siglec-7 (3–7–7), or Siglec-9 (3–9–9) (Figure 5G). An advantage of this approach is that it allowed us to use the same high affinity Sig-3L (Figure 1E) to engage each chimeric protein, thus eliminating variability due to differences in the affinity of each Siglec ligand. These proteins were transduced into Siglec-3^–/–^ cells and were expressed at similar levels (Figure S19A–D). Using the calcium flux assay to measure the response of cells preincubated with anti-TNP IgG2c and stimulated with either TNP liposomes or TNP + Sig-3L liposomes, we find that the chimeras with the cytoplasmic tails of Siglec-7 and Siglec-9 produced similar results as their full-length proteins (Figure 3d,e). Specifically, the Siglec-7 cytoplasmic tail mediated approximately 50% inhibition when engaged with ligand on the liposomes, while the Siglec-9 cytoplasmic tail mediated nearly full inhibition. Similar to Siglec-3 and -9, the cytoplasmic tail of Siglec-5 was found to inhibit FcγR activation. This is consistent with the ITIM and ITSM of Siglec-5 being nearly identical to Siglecs-3 and -9 (Figure S16).

The results from the chimera confirm that the poor inhibition of FcγR activation by Siglec-7 (Figure 5a,e) is not due to our newly discovered Sig-7L being inferior at engaging Siglec-7. In the future, development of a high affinity, selective Siglec-5 ligand would be beneficial to further confirm the role of Siglec-5 in inhibiting FcγRs. However, creation of a ligand has its own challenges as Siglec-5 has a paired receptor, Siglec-14, with nearly identical ligand binding, but opposing functions (inhibitory Siglec-5 and activatory Siglec-14), which is also found on neutrophils and monocytes.^15^ Thus, using a glycan ligand to study the ability of Siglec-5 to inhibit FcγRs would require genetic controls to ensure that there is no interference from Siglec-14.

### Assessing the Ability of Siglecs To Inhibit FcγRs in Cellular Interactions

**Figure 6:**
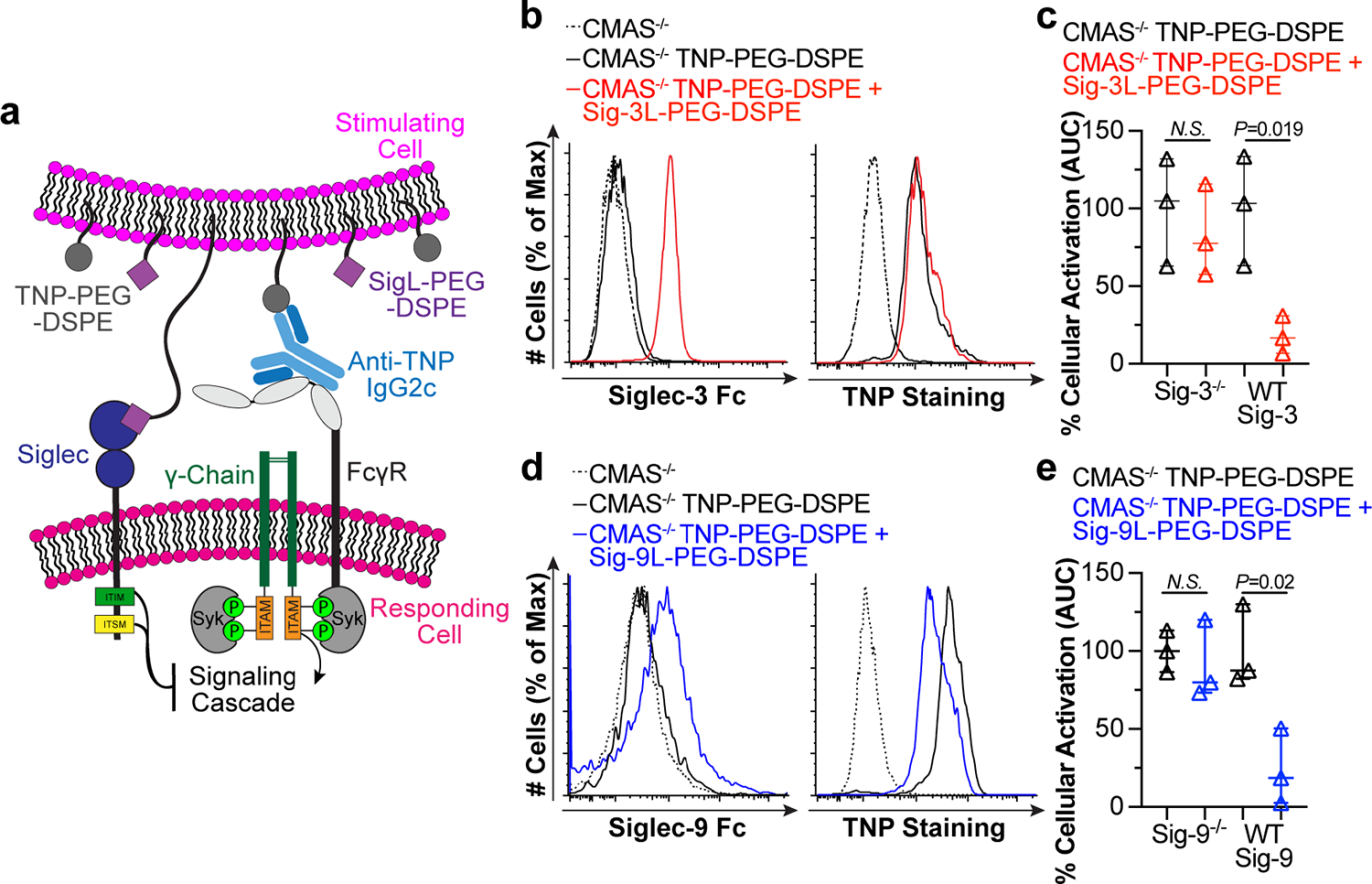
Lipid insertion of ligands into cells allows cells to coengage Siglecs and FcγRs on other cells. (a) Schematic of cell–cell stimulation assay. (b) Siglec-3 Fc staining and TNP staining in CMAS^–/–^ cells loaded with TNP–PEG–DSPE and/or Sig-3L–PEG–DSPE. (c) AUC from calcium flux of Siglec-3^–/–^ or WT Siglec-3 cells given the indicated stimulating cells. (d) CMAS^–/–^ cells loaded with TNP–PEG–DSPE and/or Sig-9L–PEG–DSPE stained with Siglec-9 Fc or TNP antibody. (e) AUC from calcium flux of Siglec-9^–/–^ or WT Siglec-9 cells given the indicated stimulating cells. In c and f, the AUC values are plotted with unloaded CMAS^–/–^ cells area subtracted and normalized to the amount of activation from TNP–PEG–DSPE loaded cells. *P* values were obtained for three technical replicates using unpaired Student’s *t* tests, and the plotted data are presented as median with 95% CI.

To test the hypothesis that Siglecs can inhibit FcγR activation through cell–cell interaction, we developed a cell–cell stimulation assay (Figure 6a). Specifically, we loaded U937 CMAS^–/–^ cells with TNP–PEG–DSPE or TNP–PEG–DSPE and Sig-3L–PEG–DSPE to serve as our “stimulating cells” (Figure 6b). U937 cells prepared for calcium flux, with or without Siglec-3, were designated as our “responding cells”. Stimulating cells could then be combined with responding cells, and cellular activation could be monitored. Using this platform, the stimulating cells behaved similar to that of liposomes, as a Siglec-3-dependent decrease in activation was observed when stimulating cells displayed the Siglec-3 ligand. Particularly, cellular activation of CMAS^–/–^ cells loaded with TNP–PEG–DSPE + Sig-3L–PEG–DSPE was reduced to baseline when compared to CMAS^–/–^ cells loaded only with TNP–PEG–DSPE (Figure 6c). This phenomenon occurred in cells expressing WT Siglec-3, but not in Siglec-3^–/–^ cells, suggesting that this effect was Siglec-3 dependent. CMAS^–/–^ cells loaded with TNP–PEG–DSPE and/or Sig-9L–PEG–DSPE (Figure 6d) also had Siglec-9-dependent inhibition of FcγR (Figure 6e). CMAS^–/–^ U937 cells, deficient in cell surface sialic acid, were used as loading cells to ensure that endogenous Siglec ligands could not engage multiple Siglecs on responding cells. Similarly, using K562 cells as the stimulating cells with U937 cells lacking Siglecs or expressing only Siglec-3 yielded comparable results (Figure S20A,B). Taken together, passive lipid insertion of FcγR ligands with Siglec-3 or -9 ligands onto cells can coengage these two receptors to reduce cellular activation.

It has been proposed that Siglecs may limit the effectiveness of tumor-targeting antibodies.^27,40^ In recent years, there has been a surge of studies examining the role of Siglec-E (murine Siglec-9) in antitumor immune responses. Notably, the expression of Siglec-E on phagocytic cells has been linked to reduced survival in patients with brain tumors.^35,37,39^ Further to this, the removal or blockade of Siglec-E has been seen to have a synergistic effect when used in combination with known immune checkpoint inhibitors such as anti-PD-1/PD-L1 therapy.^35,39^ This previous evidence of Siglecs behaving as immune-checkpoint molecules, in combination with our findings that Siglec-9 can fully inhibit FcγR-mediated immune cell activation toward cells expressing Siglec-9 ligands, demonstrates the potential for targeting Siglecs in antitumor treatments.

Antibody–lectin chimeras for immune checkpoint therapy have also been recently developed based on the premise that Siglecs decrease the effectiveness of antitumor antibodies by inhibiting FcRs.^73^ Specifically, bispecific chimeric proteins with a tumor-engaging antibody on one arm and a Siglec-Fc on the other arm were generated to engage FcRs through the antibody while simultaneously binding to Siglec ligands on tumor cells.^73^ This work is based on the idea that if Siglecs bind to ligands on the tumor cell, they can be brought together with FcRs and inhibit FcR activation, which, in turn, allows the tumor cell to evade immune detection. By binding to the chimera instead of the ligands on cancer cells, the FcR is not inhibited and can become activated toward the tumor cells. When thinking in terms of cancer immunotherapy, liposomes displaying both Siglec ligands and FcR ligands can be thought of as a tumor cell that is evading immune cell activation. Blocking this interaction under diseased conditions is a promising therapeutic strategy for future development of cancer immunotherapies.

Inappropriate activation of FcγRs can lead to various diseases;^8−11^ therefore, blocking FcγR activation has significant therapeutic potential. Siglec-independent approaches for regulating FcγRs include small molecule inhibitors of FcγRs or downstream ITAM signaling elements (e.g kinases), coengagement of activatory FcγRs with inhibitory FcγRs, administration of intravenous Ig to block FcγRs, and genetic modification.^74−77^ Coengaging an inhibitory Siglec, expressed only on select set of immune cells, with a specific FcγR allows for increased specificity and regulation of FcγRs. Previous *in vivo* experiments revealed that liposome coengagement of Siglec-3 or −8 and FcεRs reduced IgE-dependent anaphylaxis in mice.^43,55^ Our liposome platform to coengage Siglec-3 or -9 with FcγRs has the potential for dampening activation of FcγRs *in vivo*, with the caveat being that transgenic mouse models would be required due to the significant differences between human and mouse Siglecs.^78^

## Conclusions

Liposomes multivalently displaying specific Siglec ligands enabled us to study the ability of an individual Siglec to regulate FcγRs. Without enforcing their coligation, we found no evidence that Siglecs inhibit FcγRs. However, when Siglecs-3 or -9 were coengaged with FcγRs, they inhibited FcγR-mediated cellular activation. Specifically, Siglec-3 can fully inhibit FcγRI through the recruitment of SHP-1 and SHP-2 phosphatases to the ITIM. FcγRI inhibition via Siglec-9, however, occurs through the recruitment of SHP-1 and SHP-2 to both the ITIM and ITSM. Siglec-7 is highly masked by cis ligands and has an ITIM and ITSM consensus sequence that is not optimal for inhibiting FcγRI. The cytosolic motif of Siglec-5 was capable of strongly inhibiting FcγRI activation. Although this study focused on the ability of Siglecs to antagonize the specific family of FcγRs, this approach should find more widespread use for systematically studying the regulation of one immunomodulatory receptor by another.

Our cell–cell based assay involving the passive lipid insertion of FcγR ligands with and without Siglec-3/9 ligands into cells hints at the natural role of myeloid cell inhibition of FcγRs via an immunological synapse containing Siglec ligands. These findings provide experimental support for the hypothesis that Siglecs can inhibit FcγR-mediated myeloid cell activation toward tumor cells or tumor-targeting antibodies by binding to sialylated ligands on tumor cells and inhibiting FcγR activation.^27^ Thus, these findings further demonstrate that Siglecs serve as excellent targets for immune therapy and can be used for the development of cancer immunotherapies. In the future, it will be interesting to test this cell–cell-based assay in tumor cells with high sialylation to better understand the ability of Siglecs to inhibit immune responses in the context of a natural immunological synapse between myeloid cells and cancerous cells. The ability to down-regulate FcγR activation also has significant therapeutic potential in diseases where FcγRs are overactive.^8−11^ The biocompatibility and ease of modifying liposomes make liposomes an excellent option for future studies examining how Siglecs may regulate other classes of activatory receptors.

## Methods

### Cell Culture

Human U937 cell lines (ATCC) were cultured under sterile conditions at 37 °C, 5% CO_2_ in Roswell Park Memorial Institute Medium 1640, 10% fetal bovine serum (FBS) (Gibco), 100 U/mL penicillin (Gibco), and 100 μg/mL streptomycin (Gibco) (complete media). HEK 293T cells were also cultured in sterile media at 37 °C, 5% CO_2_. The media used for HEK293T cells was Dulbecco’s Modified Eagle Medium/Nutrient Mixture (DMEM) (Gibco), 10% FBS (Gibco), 100 U/mL penicillin (Gibco), and 100 μg/mL streptomycin (Gibco). Sp2/0 cells were grown in Iscoves modified Dulbecco’s Medium (IMDM) (Gibco) containing 10% FBS, 100 U/mL penicillin (Gibco), 100 μg/mL streptomycin (Gibco), and 55 μM 2-mercaptoethanol (Gibco) (complete IMDM media). Postfusion with splenocytes, developing hybridomas were selected by initial growth in complete IMDM medium containing hypoxanthine (18 μg/mL), aminopterin (176 ng/mL), and thymidine (3.8 μg/mL) HAT supplement (Corning).

### Mice

Female C57Bl/6J mice were obtained from Jackson Laboratory and bred at The Scripps Research Institute (TSRI). Animal studies were approved by the TSRI Institutional Animal Care and Use Committee.

### Site-Directed Mutagenesis

Mutations of key residues in Siglecs were performed using the megaprimer protocol from ref (79). Briefly, Siglec in a template vector (pcDNA5) was amplified through polymerase chain reaction (PCR) using a standard forward primer of the Siglec and a reverse primer containing the desired mutation. The resulting DNA, or “megaprimer” from the PCR reaction, was gel-purified and extracted using the Gel Extraction Kit (Qiagen). A second PCR reaction on the Siglec template was done using the megaprimer as a forward primer and a standard reverse primer of the Siglec. The resulting PCR reaction was again gel-purified and extracted before being digested with NheI (New England Biolabs (NEB)) and AgeI (NEB) and ligated using instant sticky-end ligase master mix (NEB) to pcDNA5 backbone also digested with NheI (NEB) and AgeI (NEB). The ligated product was transformed into DH5α competent cells (NEB) and selected for on 100 μg/mL ampicillin Luria–Bertani (LB) agar plates. Six colonies were picked, grown in LB median containing 100 μg/mL ampicillin, and then miniprepped (Qiagen) and sequenced by Sanger sequencing. A list of mutagenic primers can be found in Table S1.

### CRISPR/Cas9 Genetic Knockout

Custom crRNAs were designed to target specific genes; human Siglec-3 (target sequence = GAACACCCCCATCTTCTCC), human Siglec-5 (target sequence = GAGAGGTGGTCCGCTTCAC), human Siglec-7 (target sequence= CATGCCCTCTTGCACGGTCA), CMAS (target sequence= GAACACCCCCATCTTCTCC) (Integrated DNA Technologies; IDT). Twenty-four hours prior to transfection, 500 000 cells were plated in a 12-well tissue culture plate. Then 1 μM crRNA and 1 μM ATTO-550 labeled tracrRNA (IDT) were combined and heated at 95 °C for 5 min to create 1 μM gRNA. To each well in a 12-well plate, 1.2 μg of gRNA, 6.25 μg of Cas9 nuclease (IDT), 12.5 μg Cas9 Plus reagent (IDT), and 7.5 μL CRISPRMAX reagent (Thermo Fisher) in 250 μL Opti-MEM medium (Gibco) were added. After 24 h incubation period at 37 °C, 5% CO_2_, cells were washed, resuspended in 400 μL of sterile flow cytometry staining buffer (FACS) (Hank’s Balanced Salt Solution pH 7.4 containing 1% FBS and 500 μM EDTA), and stored on ice until sorting. Cells were sorted within the University of Alberta Flow Cytometry Core. The top 5% of cells fluorescing ATTO-550 were sorted into four 96-well flat-bottom plates at one or three U937 cells per well. Cells were grown for approximately 3 weeks until there were enough cells to screen expression by flow cytometry using phycoerythrin (PE)-conjugated antibodies. Clones staining negative for targeted Siglec were grown and sequence validated. This required PCR amplifying the DNA fragment flanking the target sequence and Sanger sequencing the sample, also done within the University of Alberta Biological Services Core.

### Production of Anti-TNP-IgG

Female C57BL/6J mice were immunized intraperitoneally with 20 μg of TNP:KLH (15:1) emulsified in 100 μL of Imject incomplete Freund’s adjuvant (Thermo Scientific). At 4 and 10 weeks, animals were boosted intraperitoneally with 5 μg in 100 μL of PBS, before splenocytes were isolated in PBS and fused with Sp2/0 cells using Hybri-max PEG/DMSO (Sigma). TNP reactive hybridomas were selected in HAT medium, screened by enzyme-linked immunosorbent assay (ELISA) using TNP:BSA coated plates (5 μg/mL), and then cloned by limiting dilution. Selected hybridomas were cultured to high density in Iscove’s Modified Eagle Medium ((IMDM), 1% FBS (Gibco)), and antibody was isolated by Protein G chromatography. Bound antibody was eluted with glycine pH 2.8, neutralized with 100 mM Tris pH 8, and then dialyzed against PBS.

### Siglec Expression on Neutrophils from Whole Blood

Three milliliters of human blood was lysed using RBC lysis buffer (1.5 M ammonium chloride (Sigma-Aldrich), 100 mM potassium hydrogen carbonate (Sigma-Aldrich), 1 mM ethylenediamine tetraacetic acid (EDTA) (Sigma-Aldrich)) for 5–7 min and then centrifuged at 300 rcf for 5 min. A second lysis was performed for 3–5 min and spun again at 300 rcf for 5 min. The red blood cells were resuspended in 1:100 of Human TruStain FcX Fc receptor blocking solution (Biolegend) to FACS for 10 min at room temperature. PE-labeled α-human Siglec antibodies/isotype controls (Biolegend and BD Biosciences) (1:25), α-human CD14 (1:500), and α-human CD15 (1:500) were directly added to the cells. After a 20–30 min incubation at 4 °C, cells were washed and resuspended in 1:1000 propidium iodide to FACS buffer. Flow cytometry was carried out on a LSR Fortessa flow cytometer (BD) in the PE, PE-Texas Red, BUV395, and BV605 channels. A more detailed list of antibodies used can be found in Table S3.

### Antibody Binding Assays

U937 cells were incubated with 1:100 of Human TruStain FcX Fc receptor blocking solution (Biolegend) to FACS for 10 min at room temperature. PE-labeled α-human antibodies (Biolegend and BD Biosciences) or isotype controls (Biolegend) were directly added to the cells in a 1:125 ratio of antibody to FACS buffer to yield a final antibody concentration of 1:250. See Table S3 for the details regarding the clones and catalogue number of each antibody used. After a 25 min incubation at 4 °C, the cells were washed and resuspended in FACS buffer. Flow cytometry was carried out on a LSR Fortessa flow cytometer (BD) in the PE channel.

### Liposome Formulation

All liposomes were made using a 57:38:5 molar ratio of distearoylphosphatidylcholine (DSPC) (Avanti Polar Lipids), cholesterol (Sigma-Aldrich), and polyethylene glycol-2000-distearoyl phosphoethanolamine (PEG–DSPE) (Avanti Polar Lipids). Ligands conjugated to PEG–DSPE were included in the 5% PEG–DSPE. To assemble the liposomes, chloroform solutions of DSPC, cholesterol and PEG–DSPE were mixed together. Excess chloroform was evaporated using N_2_ (g) and DMSO solutions of ligand–PEG–DSPE were added to the dried liposome mixture. The liposomes were lyophilized overnight then hydrated in PBS, pH 7.4 (Corning) to achieve a final liposome concentration of 1–2 mM. To get the liposome mixtures into solution, the mixtures were sonicated in a water bath 3× for 30 s. Liposomes were extruded at room temperature using a miniextruder (Avanti Polar Lipids) through polycarbonate membrane filters with 400 and 100 nm pore sizes (Millipore) 20 times each. Liposomes for functional assays were purified over a Sepharose CL-4B column (Sigma-Aldrich) upon extrusion.

### Liposome Binding Assays

Approximately 75 000 cells/well were plated in a 96-well U-bottom cell culture plate. Fluorescent liposomes, containing 0.1% Alexa Fluor-647–PEG–DSPE were added to cells in media to give a final concentration of 50 μM. The cells were incubated at 37 °C for 1 h, washed, and resuspended in FACS buffer. Flow cytometry was carried out on a LSR Fortessa flow cytometer (BD) in the APC channel.

### Production of Lentiviral Vectors

The previously described lentiviral backbone, RP172, was used to make all lentiviral vectors.^80^ PCR, using the primers in Table S2, was used to install SphI and PacI restriction sites surrounding the viral vector. The resulting DNA could be digested using SphI (NEB) and PacI (NEB) and ligated using instant sticky-end ligase master mix (NEB) to RP172 backbone also digested with SphI (NEB) and PacI (NEB). Ligated vectors were then transformed into stable competent *E*. *coli* (NEB), to decrease plasmid instability, and selected for on 100 μg/mL ampicillin LB agar plates at 37 °C. A period of 16–24 h later, six clones were picked and transferred into LB media containing 100 μg/mL ampicillin for another 24 h at 37 °C. Following minipreps (Qiagen), the DNA sequence of the lentiviral vector was confirmed using Sanger sequencing.

### Production and Transduction of Lentivirus

Lentivirus was produced following our previous protocol.^81^ Briefly, approximately 900 000 HEK293T cells were plated in a six-well dish with 1.5 mL of DMEM growth medium (Gibco) containing 10% fetal bovine serum (FBS; Gibco), 100 U/mL penicillin (Gibco), and 100 μg/mL streptomycin (Gibco). The following day, 150 ng of a packaging vector (RP18), 150 ng of an envelope vector (RP19), and 300 ng of the lentiviral vector of interest were triple transfected into the HEK293T cells using TransIT-LT1 Reagent (Mirus Bio) according to the manufacturer’s protocol. Cells were incubated with this transfection mixture at 37 °C and 5% CO_2_ for 72 h. Following transfection, the supernatant was collected, spun at 300 rcf at 4 °C for 5 min, and incubated at 4 °C in Lenti-X Concentrator (Clontech) for 1 h. The supernatant solution was spun at 1500 rcf for 45 min at 4 °C. The final pellet was resuspended in one-tenth of the original volume of medium using sterile PBS and stored at −80 °C. For transduction, approximately 150 000 cells were seeded in quadruplicate in a 24-well plate in 250 μL of growth media. A range of concentrated virus −1, 2, 5, or 10 μL was added to each well and incubated at 37 °C, 5% CO_2_ for 6–8 h. After incubation, 500 μL of fresh media was added to each well and the transduction was left at 37 °C, 5% CO_2_ for 72 h. Post-transduction, the cells were harvested and resuspended in FACS buffer, and the viral titer was measured on a flow cytometer by examining the percentage of mAmetrine^+^ cells in each well. Cells with an infection (mAmetrine^+^ population) close to 1% were grown under 300 μg/mL zeocin (Invitrogen) selection until the mAmetrine^+^ population was ≥95%.

### Calcium Flux Assay for Fcγ Receptors

A 12.5 ng/mL solution of interferon-gamma (IFNγ) was added to approximately 6 million cells to differentiate the cells into more monocyte-like cells and up-regulate Fcγ receptors on the cell surface. Twenty-four hours after the addition of IFN γ, the cells were harvested and resuspended in calcium flux loading buffer (500 mL RPMI, 10 mM *N*-2-hydroxyethylpiperazine-*N*-2-ethanesulfonic acid, pH 7.2–7.5 (HEPES) (Gibco), 1% FBS (Gibco), 100 U/mL penicillin (Gibco), 100 μg/mL streptomycin (Gibco), 1 mM EDTA, and 1 mM magnesium chloride (MgCl_2_)) with 1 μM of Indo-1-acetoxymethyl ester (Indo-1-Am) (Invitrogen). Cells were incubated at 37 °C for 40 min (mixing every 10 min), washed with calcium flux loading buffer, and then resuspended in calcium flux running buffer (500 mL HBSS, 1% FBS, 1 mM MgCl_2_, 1 mM calcium chloride (CaCl_2_) with 12.5 μg/mL anti-TNP-IgG2c. After a 30 min, 4 °C incubation period, cells were thoroughly washed twice with calcium flux running buffer prior to flow cytometry. Cells were resuspended at approximately 1 million cells/mL and aliquoted (500 μL) into flow tubes. The tubes were warmed at 37 °C for 5 min prior to putting the cells on the flow cytometer and beginning calcium flux measurements. Cell stimulation with TNP liposomes, TNP-BSA, or loaded cells began 10 s into acquisition. The change in Indo-1-Am fluorescence from violet to blue was monitored by flow cytometry for 4 min at 37 °C. Data were analyzed using FlowJo (kinetics function) and Prism (plotting area under curve) software.

### CFSE Labeling

For examining the calcium flux of two cell lines simultaneously, the cells were washed with HBSS and then resuspended in 100 nM of carboxyfluorescein succinimidyl ester (CFSE) dye. After a 7 min incubation at room temperature, the reaction was quenched by the addition of RPMI Media (10% (FBS) (Gibco), 100 U/mL penicillin (Gibco), and 100 μg/mL streptomycin (Gibco)). After one wash in RPMI, the two cell lines were combined and prepared for calcium flux as described above.

### Western Blotting

U937 cells (≈ 3 × 10^6^) pretreated with 12.5 ng/mL of IFNγ for 24 h were incubated with 12.5 μg/mL anti-TNP-IgG2c in calcium flux running buffer at 4 °C for 25 min. After two washes with calcium flux running buffer, the cells were warmed at 37 °C for 5 min, 40 μL of 1 mM liposomes were added to 500 μL of cells, and the cells were incubated for 2 min at 37 °C. Ice cold PBS was then added to the cells to stop stimulation. After centrifuging the cells at 3000 rcf for 30 s, the cell pellet was resuspended in cell lysis buffer (10X lysis buffer (cell signaling), 1 mM phenylmethylsulfonyl fluoride (PMSF) (Sigma-Aldrich)) and incubated at 4 °C for 60 min. After the lysis, cells were spun at 10 000 rcf for 10 min and the supernatant was stored at −20 °C. Twenty microliters of protein sample and 4 μL of 6X loading dye (62.5 mM Tris-HCl, 2% (w/v) sodium dodecyl sulfate, 10% glycerol, 0.01% (w/v) bromophenol blue, 1.25 M DTT)) were loaded into a 12% precast gel (ThermoFisher) and run at 150 V for 1 h. Transfer of the gel onto a nitrocellulose membrane with 0.2 μm pore size (Life Technologies) was done according to miniblot module protocol (ThermoFisher). The module was run in transfer buffer (28.8 g glycine, 6.04 g Tris base, 1.6 L dH_2_O, 400 mL MeOH) at 10 V for 75 min. After transfer, the membrane was blocked overnight at 4 °C in blocking buffer (Intercept Blocking Buffer (LI-COR)). Primary antibody (3 mL of phosphate-buffered saline with 0.1% tween (PBS tween), 3 mL of intercept blocking buffer (LI-COR), 3 μL of mouse anti-Akt (Cell Signaling), 3 μL of rabbit anti-pSer473 Akt (Cell Signaling)) was added to the membrane for 2 h at room temperature. The membrane was washed with PBS tween three times for 20 min. Secondary antibody (3 mL of PBS tween, 3 mL of intercept blocking buffer (LI-COR), 0.6 μL of goat antirabbit 800 nm IRdye (LI-COR), 0.6 μL of goat ant-mouse 680 nm IR dye (LI-COR)) was added to the membrane and rotated for 1 h at room temperature. Again, the membrane was washed three times for 20 min in PBS tween and 20 min in PBS before visualizing on an Odyssey imager (LI-COR). The resulting data files were processed using ImageStudio Lite software.

### Imaging Flow Cytometry

Three million cells were pretreated with 12.5 ng/mL of IFNγ for 24 h were incubated with 12.5 μg/mL anti-TNP-IgG2c in calcium flux running buffer at 4 °C for 25 min. The cells were warmed at 37 °C for 5 min, and 50 μM liposomes were added and incubated for 2 min at 37 °C. Ice cold PBS was then added to the cells to stop stimulation. After centrifuging the cells at 3000 rcf for 30 s, BD Cytofix/Cytoperm solution was added to the cells for 5 min at room temperature and then 10 min at 4 °C. Following fixation, cells were spun at 660 rcf for 5 min and washed twice with FACS buffer. The cells were then frozen in 90% FBS, 10% DMSO until staining. For measuring Siglec colocalization with phosphatases, the cells were thawed, washed with FACS, and resuspended in 1:100 of rabbit anti-SHP-1 (Cell Signaling) or rabbit anti-SHP-2. After a 25 min incubation at 4 °C, the cells were washed and then resuspended in 1:200 of secondary antibody solution (antihuman Siglec-fluorescein-5-isothiocyanate (Biolegend) and donkey antirabbit IgG-Alexa Fluor 647 (Biolegend)) for 25 min at 4 °C. Each sample was run on an ImageStreamX Mk II flow cytometer (excitation lasers 488 and 642 nm, 60× magnification). Data analysis was performed using IDEAS software, version 6.2. For measuring Siglec colocalization with FcγRI, cells were thawed, washed with FACS, and resuspended in 1:200 solution of antihuman Siglec-3-allophycocyanin and antihuman CD64-PE for 25 min at 4 °C. Again, samples were run on an ImageStreamX Mk II Flow cytometer (excitation lasers 488 and 642 nm, 60× magnification). Data analysis was performed using IDEAS software, version 6.2.

### Fc Block of Calcium Flux

Cells were stained with Indo-1-Am dye as described above. Before staining with anti-TNP-IgG, cells were preincubated with 0.05 mg/mL of human trustain FcX Fc Receptor blocking solution (Biolegend), purified antihuman CD64 antibody clone 10.1 (Biolegend), or CD32 monoclonal antibody clone 6C4 (Invitrogen) for 10 min at room temperature. The remainder of the calcium flux was performed as described above.

### Neuraminidase Treatment of Cells

About two million cells were resuspended in 1000 μL of PBS with or without 50 μL of 0.3 mg/mL Neuraminidase A from *Arthrobacter ureafaciens* and shaken at 37 °C for 1 h. The cells were then washed with RPMI Media (10% (FBS) (Gibco), 100 U/mL penicillin (Gibco), and 100 μg/mL streptomycin (Gibco) before liposome binding assays were performed as described above.

### Linking TNP–ϵ-Aminocaproyl-OSu to BSA

TNP–ϵ-aminocaproyl-OSu was linked to bovine serum albumin (BSA) using a 1:20 ratio of 0.1 M TNP–ϵ-aminocaproyl-OSu (in 68.6 μL od dimethylformamide) to 0.1 M BSA (in 500 μL of NaHCO_3_). The scheme for this coupling can be found in Figure S21. The mixture was rotated in the dark, at room temperature, for 2 h. Unconjugated TNP-ε-aminocaproyl-OSu was removed from the solution using an Amico Ultra 30 kDa centrifugal filter unit. The amount of TNP units linked to BSA was verified by testing the absorbance at 280 and 348 nm using TNP and BSA extinction coefficients of 15 400 and 43 824, respectively.

### Ligand Conjugation to PEG–DSPE

NHS coupling of NH_2_–PEG_(45)_-DSPE to NHS-trinitrophenyl was performed as described in the Supporting Information (Figure S22). Conjugation of NHS-PEG_(45)_-DSPE to Siglec-3 ligand–ethylamine, Siglec-7 ligand–ethylamine, and Siglec-9 ligand–ethylamine was performed following the literature method.^51,52^ Schematics of these syntheses are shown in the Supporting Information (Figures S23–S25).

### Lipid Insertion into Cells

A solution of 12.5 ng/mL interferon-gamma (INFγ) was added to approximately 6 million cells 24 h prior to lipid insertion. Cells were then washed with PBS twice. A 10 μM solution of TNP–PEG–DSPE and/or Sig-3L–PEG–DSPE was added to 500 μL of cells in PBS and left to passively diffuse into the cell membrane through incubation at 37 °C for 2 h. Cells were washed once with PBS and then prepared for calcium flux.

### TNP Antibody Staining of TNP–PEG–DSPE-Loaded Cells

Lipid inserted cells (or control cells) were plated in a 96-well U-bottom plate and centrifuged at 300 rcf for 5 min. A 2.5 mg/mL solution of mouse anti-TNP-IgG2b antibody was added to half of the cell pellets in a 1:200 ratio of antibody:FACS buffer, and the cells were incubated at 4 °C for 25 min. The cells were spun at 300 rcf for 5 min and then resuspended in 1:100 goat antimouse IgG2b-AF647 (ThermoFisher, catalog no. A-21242):FACS buffer for 25 min at 4 °C. After incubation, cells were centrifuged and resuspended in 150 μL of FACS buffer. Flow cytometry was carried out on a LSR Fortessa flow cytometer (BD), examining the presence of TNP on the cell surface by looking at differences in the AF647 (APC) channel.

### Precomplexing Liposome with Anti-TNP-IgG2c

A 3.3 μL solution of 0.6 mg/mL of anti-TNP-IgG2c was added to 40 μL of 1 mM liposome and mixed well. The liposome–antibody solution was incubated at 4 °C for 30–40 min. The resulting precomplexed liposome could then be used in Ca^2+^ flux assays or liposome binding assays. At 0.1 mol % of TNP in 1 mM liposome solution, we can estimate a concentration of 0.5 μM TNP in solution assuming that 50% of TNP ends up on the outer surface of the liposome. For the titration of antibody, 0.16 μM (1:0.32 TNP:antibody), 0.33 μM (1:0.66), 0.66 μM (1:1.32), 1.33 μM (1:2.66), and 2.66 (1:5.32) of 0.6 mg/mL anti-TNP-IgG2c were used.

### Siglec Fc Production and Staining

Production of Siglec-3 Fc and the flow cytometry staining were performed as previously described.^68^

## Supporting Information Available

Further details supporting the main manuscript. Primers for PCR; antibodies for flow cytometry; Siglec expression; cell line characterization; antibody development; trinitrophenyl liposome binding; Fc receptor expression; Siglec intrinsic inhibition; additional calcium flux experiments; precomplexed liposome stimulation; Erk Western blots; imaging flow colocalization; imaging flow SHP-1, SHP-2, SHIP-1, and SHIP-2 recruitment; Siglec sequencing alignment; Siglec-7 ligand binding; Siglec-3 chimera characterization; cell–cell stimulation with K562 cells; trinitrophenyl conjugation to bovine serum albumin; ligand conjugation to lipid (PDF)
Transparent Peer Review report available (PDF)
