# Supplementary material for: Dissecting the Ability of Siglecs To Antagonize Fcγ Receptors
Source: ACS Cent Sci. 2024 Jan 17;10(2):315–30. doi: 10.1021/acscentsci.3c00969 (PMC10906256; doi:10.1021/acscentsci.3c00969)
Supplement: Supplementary file 1 — oc3c00969_si_001.pdf [file oc3c00969_si_001.pdf]

Supplementary Materials

Dissecting the ability of Siglecs to antagonize Fcg receptors

Kelli A. McCord[1], Chao Wang[2], Mirjam Anhalt[1], Wayne W. Poon[3], Amanda L. Gavin[4], Peng Wu[2], and Matthew S. Macauley[1,5,*]

[1] Department of Chemistry, University of Alberta
[2] Department of Molecular Medicine, Scripps Research Institute
[3] Institute for Memory Impairments and Neurological Disorders, University of California
[4] Department of Immunology and Microbiology, Scripps Research Institute
[5] Department of Medical Microbiology and Immunology, University of Alberta

[*] email: macauley@ualberta.ca

Table of Contents

Table S1. Forward (FWD) and Reverse (RVS) Site-Directed Mutagenesis Primers. S4

Table S2. Viral vector primers to install SphI and PacI. S4

Table S3. Antibodies used in flow cytometry staining of cells. S5

Figure S1. Siglec expression on primary peripheral blood human neutrophils and monocytes and U937 cells. S6

Figure S2. Creation of cell lines. S7

Figure S3. Development of mouse monoclonal anti-trinitrophenyl IgG antibodies. S8

Figure S4. Trinitrophenyl (TNP) conjugation to PEG-DSPE. S9

Figure S5. Optimization of FcγR dependent TNP-liposome binding to cells. S10

Figure S6. FcγR expression on human monocytes derived from primary peripheral blood. S11

Figure S7. Siglecs do not intrinsically inhibit FcγR activation when stimulated with liposomes. S12

Figure S8. Three independent experiments showing Siglec-3, -9, and -7 inhibition of FcγRs. S13

Figure S9. Creation of cells expressing Siglecs with the critical arginine residue required for binding mutated. S14

Figure S10. Calcium flux of Siglec-3 and FcγR engagement on separate liposomes or WT cells. S15

Figure S11. Liposomes pre-complexed with anti-TNP antibody bind and stimulate U937 cells. S16

Figure S12. Phosphorylation of downstream protein Erk is decreased when Siglecs-3 or -9 are co-engaged with FcγRs. S17

Figure S13. Imaging flow cytometry examining liposome, FcγR, and Siglec-3 colocalization. S18

Figure S14. SHP-2 recruitment to Siglec-3 and -9. S19

Figure S15. SHIP-1 and SHIP-2 recruitment to Siglec-3 and -9 by imaging flow cytometry. S20

Figure S16. Sequencing alignment of Siglec-3, -5, -7 and -9 cytosolic tails. S21

Figure S17. Removal of sialic acid from the cell surface increases Sig-7L liposome binding. S22

Figure S18. Creation of cells expressing Siglec-7 cytosolic mutants. S23

Figure S19. Creation of Siglec-3 chimera proteins. S24

Figure S20. Lipid insertion of TNP-PEG-DSPE and/or Sig-3L-PEG-DSPE into K562 cells. S25

Figure S21. Trinitrophenyl conjugation to Bovine Serum Albumin. S26

Figure S22. TNP-NHS conjugation to NH_2-$PEG_{(45)}$-DSPE. S27

Figure S23. Siglec-3 ligand with an ethylamine conjugation to NHS-$PEG_{(45)}$-DSPE. S28

Figure S24. Siglec-7 ligand with an ethylamine conjugation to NHS-$PEG_{(45)}$-DSPE. S29

Figure S25. Siglec-9 ligand with an ethylamine conjugation to NHS-$PEG_{(45)}$-DSPE. S30

Table S1. Forward (FWD) and reverse (RVS) site-directed mutagenesis primers.

Mutation Site	Primers
Y433F Siglec-9	FWD: 5'– GGAAGGAGAGCTCCAGTTTGCATCCCTCAGCTTCC –3'<br>RVS: 5'– GGAAGCTGAGGGATGCAAACTGGAGCTCTCCTTCC –3'
Y456F Siglec-9	FWD: 5'– GGCCACTGACACCGAGTTTTCGGAGATCAAGATCC –3'<br>RVS: 5'– GGATCTTGATCTCCGAAAACTCGGTGTCAGTGGCC –3'
R120A Siglec-9	FWD: 5'– GCGGGGAGATACTTCTTTGCTATGGAGAAAGGAAGTATAAAATG –3'<br>RVS: 5'– CATTTTATACTTCCTTTCTCCATAGCAAAGAAGTATCTCCCCGC –3'
P439S Siglec-7	FWD: 5'– CCAGTATGCATCCCTCAGCTTTCATAAG –3'<br>RVS: 5'– CTTATGAAAGCTGAGGGATGCATACTGG –3'
N458T Siglec-7	FWD: 5'– GAAGCCACCAACACTGAGTACTCAGAG –3'<br>RVS: 5'– CTCTGAGTACTCAGTGTTGGTGGCTTC –3'
3-5-5 Chimera	FWD: 5'– GCAGTATTAATTAAGCCACCATGCCGCTGCTGCTACTG –3'<br>RVS I: 5'– CCATGACACCAGCACCACCATGAACCACTCCTGCTC –3'<br>RVS II: 5'– TAACGAGCATGCTCACTTGCTTGTCTTGATC –3'
3-7-7 Chimera	FWD: 5'– GCAGTATTAATTAAGCCACCATGCCGCTGCTGCTACTG –3'<br>RVS I: 5'– CCCAGCAACACTCCTGATACATGAACCACTCCTGCTCTGGT –3'<br>RVS II: 5'– TAACGAGCATGCTCACGTTACAATCTCACTATAT –3'
3-9-9	FWD: 5'– GCAGTATTAATTAAGCCACCATGCCGCTGCTGCTACTG –3'<br>RVS I: = 5'– CTCCAGCTCCCCCGACCAATGAACCACTCCTGCTCTGG – 3'<br>RVS II: 5'– TAACGAGCATGCTCATCTGTGGATCTTGATCTCCG –3'

Table S2. Viral vector primers to install SphI and PacI.

Mutation Site	Primers
Siglec-5 Viral FWD	5' – GCAGTATTAATTAAGCCACCATGCTGCCCCTGCTGCT – 3'
Siglec-5 Viral RVS	5' – TAACGAGCATGCTCACTTGCTTGTCTTGATCTCC – 3'
Siglec-7 Viral FWD	5' – GCAGTATTAATTAAGCCACCATGCTGCTGCTGCTGCT – 3'
Siglec-7 Viral RVS	5' – TAACGAGCATGCTACTTGGGGATCTTGATCTCT – 3'
Siglec-9 Viral FWD	5' – GCAGTATTAATTAAGCCACCATGCTGCTGCTGCTGCT – 3'
Siglec-9 Viral RVS	5' – TAACGAGCATGCTCATCTGTGGATCTTGATCTCCG – 3'

Table S3. Antibodies used in flow cytometry staining of cells.

Antibody	Clone	Catalogue Number	Company
PE Anti-hFcγRI	10.1	305007	BioLegend
PE Anti-hFcγRII	FUN-2	303205	BioLegend
PE Anti-hFcγRIII	3G8	302007	BioLegend
PE Anti-hSiglec-1	7-239	346004	BioLegend
PE Anti-hSiglec-2	HIB22	302506	BioLegend
PE Anti-hSiglec-3	WM53	303404	BioLegend
PE Anti-hSiglec-5	1A5	352004	BioLegend
PE Anti-hSiglec-6	-	FAB2859P	R&D
PE Anti-hSiglec-7	6-434	339204	BioLegend
PE Anti-hSiglec-8	7C9	347104	BioLegend
PE Anti-hSiglec-9	K8	351504	BioLegend
PE Anti-hSiglec-10	5G6	347604	BioLegend
Anti-hSiglec-11	4C4	681702	BioLegend
PE Mouse IgG2b, κ isotype	27-35	402204	BioLegend
PE Mouse IgG1, κ isotype	MOPC-21	400139	BioLegend
Human TruStain FcX™	-	422302	BioLegend
Anti-hFcγRI	10.1	305002	BioLegend
Anti-hFcγRII	6C4	16-0329-81	Invitrogen
BV605 Anti-hCD14	M5E2	301834	BioLegend
BUV395 Anti-hCD15	HI98	563872	BD Biosciences

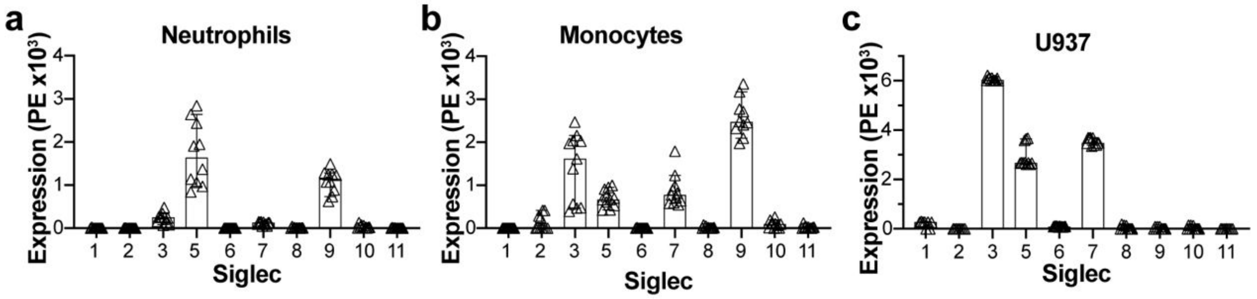

Figure S1. Siglec expression on primary peripheral blood human neutrophils and monocytes and U937 cells. (a,b,c) Flow cytometry staining of Siglecs-1,- 2, -3, -5, -6, -7, -8, -9, -10, and -11 in neutrophils (a) and monocytes (b) isolated from human blood as well as U937 WT cells (c). Values are plotted as mean fluorescence intensity (MFI) of anti-Siglec-phycoerythrin (PE) signal with isotype control subtracted. Ten biological replicates were plotted and the error bars on a, b, and c data plots are presented as median with 95% confidence interval (CI).

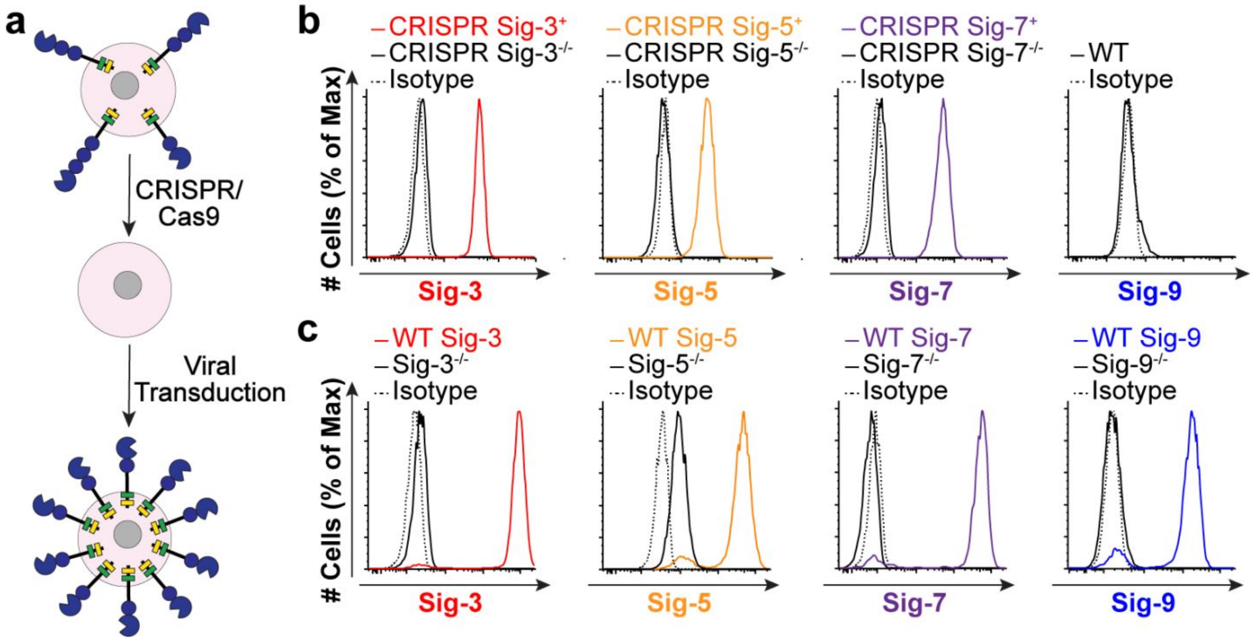

Figure S2. Creation of cell lines. (a) Schematic illustrating the deletion of a Siglec of interest from U937 cells using CRISPR/Cas9 followed by the viral transduction of the cells with empty vector (Siglec$^{-/-}$) or WT Siglec. (b) Flow cytometry histograms illustrating the staining for the presence of a Siglec after CRISPR/Cas9 genetic knockout. (c) Expression of Siglec in CRISPR/Cas9 cells after viral transduction of empty lentiviral vector (Siglec$^{-/-}$) or WT Siglec as determined by flow cytometry staining.

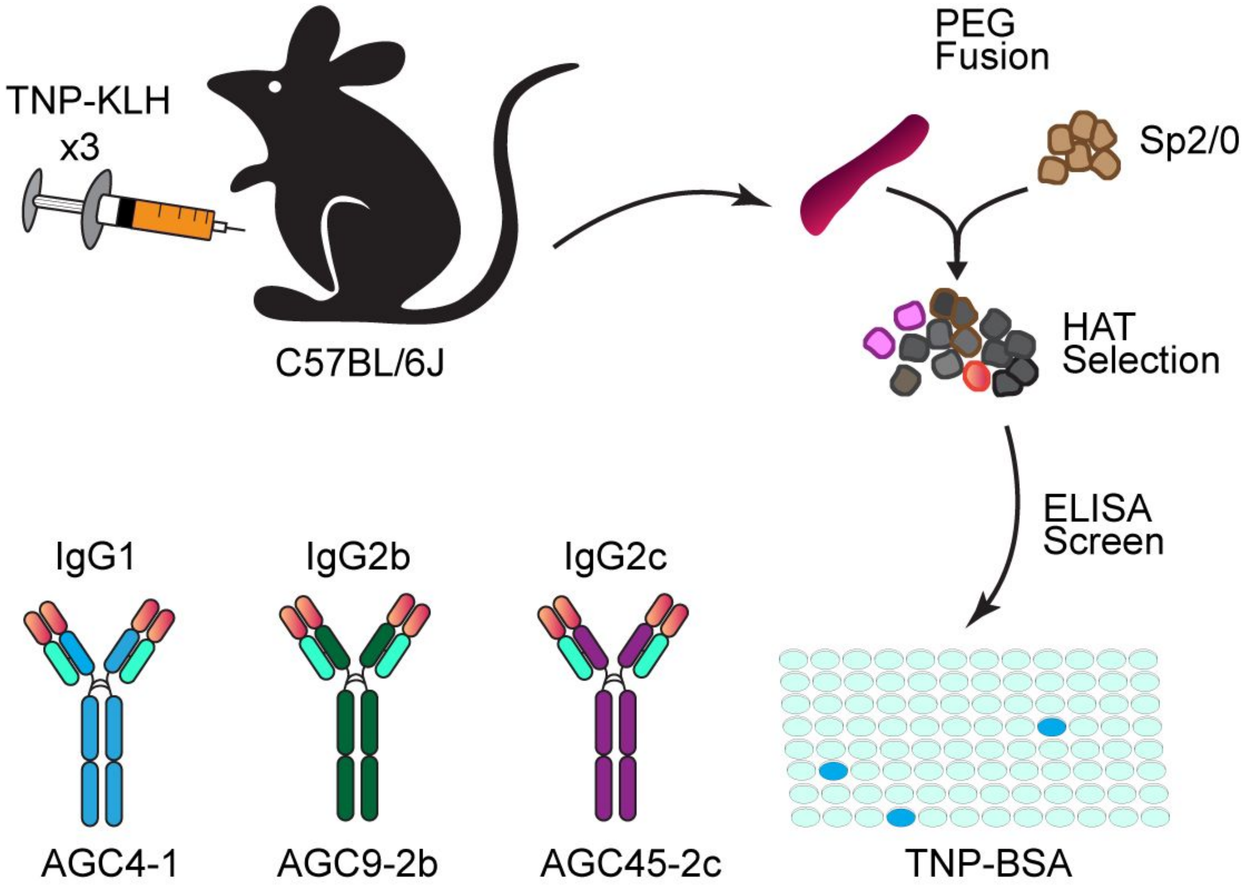

Figure S3. **Development of mouse monoclonal anti-trinitrophenyl IgG antibodies.** C57BL/6J mice were repeatedly immunized with TNP:KLH intraperitoneally before total splenocytes were isolated and fused with Sp2/0 cells using PEG. HAT resistant hybridomas were screened for secretion of TNP specific antibody by ELISA with TNP:BSA coated plates. TNP specific hybridomas of different isotopes were isolated and cloned by limiting dilution.

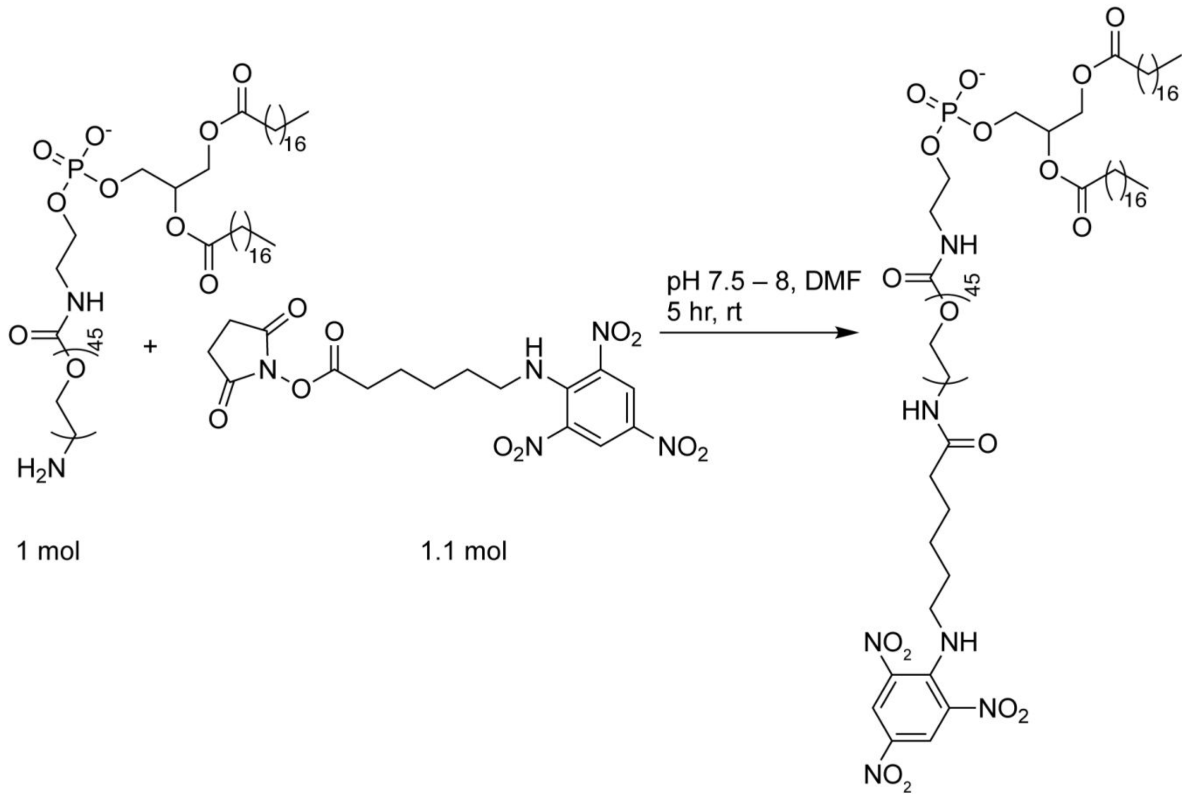

Figure S4. Trinitrophenyl (TNP) conjugation to PEG-DSPE. Conjugation of NHS-trinitrophenyl to NH_2-PEG_{45}-DSPE was done using 1 equivalent of NH_2-PEG_{45}-DSPE mixed with 1.1 equivalent of NHS-trinitrophenyl. These two compounds were mixed together in dried dimethylformamide (DMF) at a pH between 7.5-8 for 5 hr at room temperature.

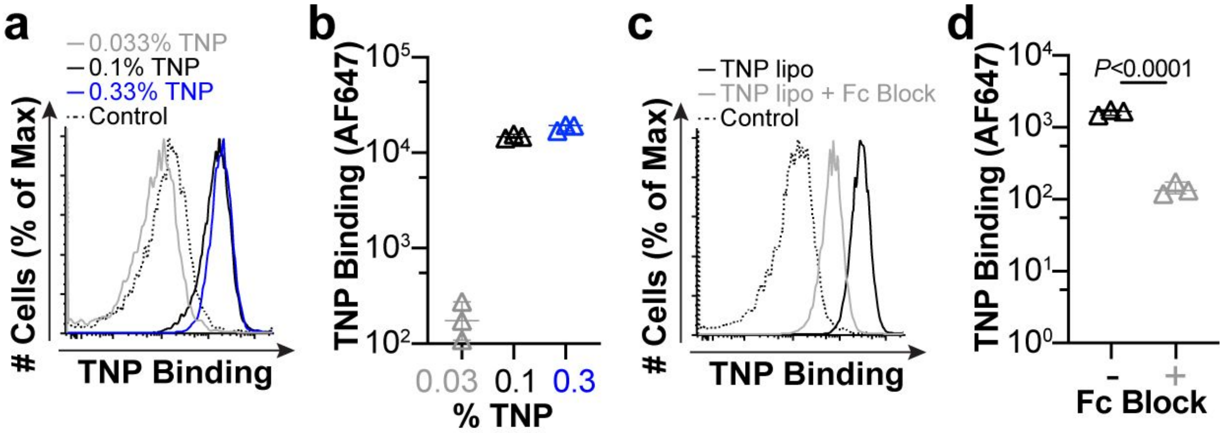

Figure S5. Optimization of FcγR dependent TNP-liposome binding to cells. (a,b) Raw flow cytometry histograms (a) and plotted MFI quantification (b) of liposomal nanoparticles loaded with 0, 0.03, 0.1, or 0.3 mol % of TNP-PEG-DSPE with 0.1 mol % AF647-PEG-DSPE binding to cells pre-incubated anti-TNP-IgG2c. Binding (MFI) values from a control liposome bearing only 0.1 mol % AF647-PEG-DSPE were subtracted from the plotted MFI values. (c,d) TNP-liposome binding histogram (c) and MFI quantification (d) to cells pre-treated with anti-TNP-IgG2c with or without Fc block. Data plots in b and c are presented as median with 95% CI. Statistical analysis was performed on three technical replicates using an unpaired Student's t-test.

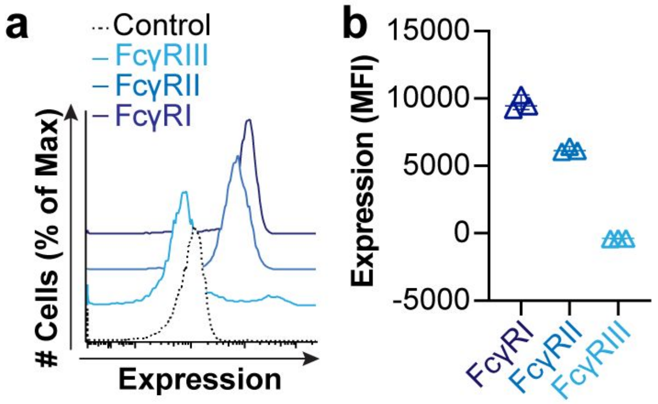

Figure S6. FcγR expression on human monocytes derived from primary peripheral blood. (a) Flow cytometry representative histograms of FcγRI, FcγRII, FcγRIII expression on monocytes isolated from human blood. (b) Quantification of three technical replicates MFI values plotted. Error bars are presented as median with 95% CI.

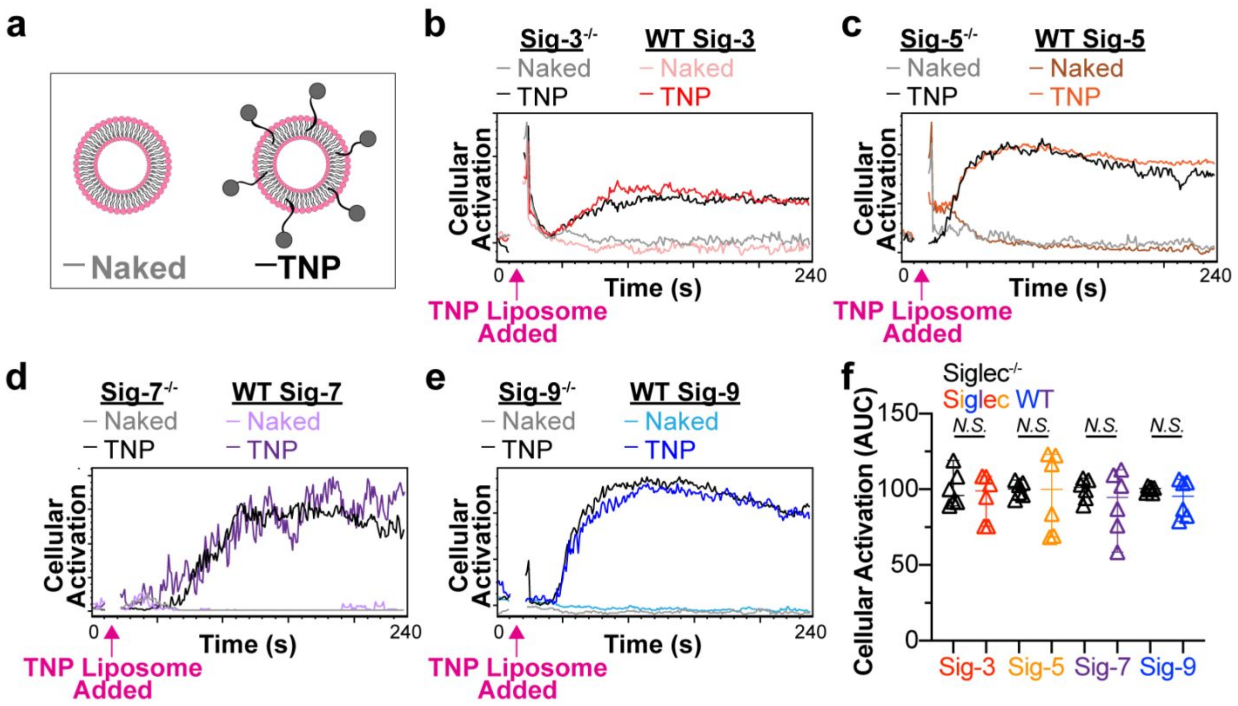

Figure S7. Siglecs do not intrinsically inhibit FcγR activation when stimulated with liposomes. (a) Schematic of liposomes used to measure overall activation of cells. (b,c,d,e) Representative calcium flux of Siglec-3$^{-/-}$ and WT Siglec-3 (b), Siglec-5$^{-/-}$ and WT Siglec-5 (c), Siglec-7$^{-/-}$ and WT Siglec-7 (d), and Siglec-9$^{-/-}$ and WT Siglec-9 (e) after administration of the indicated liposomes. Cells were loaded with INDO-1-AM followed by an anti-TNP-IgG2c antibody. The pink arrow represents addition of the liposome ten seconds after acquisition on the flow cytometer. (f) Area under the calcium flux curves (AUC) from TNP liposome administration plotted with naked liposome AUC subtracted in Siglec$^{-/-}$ cells (Black) or WT Siglec cells (colour). Values are normalized to the amount of TNP liposome activation in Siglec$^{-/-}$ cells. Error bars are presented as median with 95% CI and P values were determined using unpaired Student's t-tests for six technical replicates per condition.

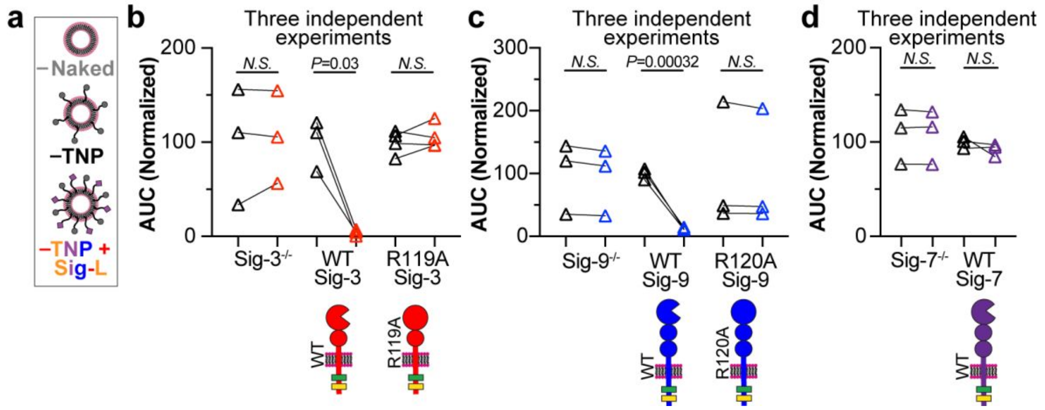

Figure S8. Three independent experiments showing Siglec-3, -9, and -7 inhibition of FcγRs. (a) Liposomes used for stimulation of cells. (b,c,d) Area under the calcium flux curve (AUC) after administration of the indicated liposomes to Siglec-3 cell lines (b), Siglec-9 cell lines (c), and Siglec-7 cell lines (d). Each data point represents an average of three technical replicates and each pair of data points was taken on three different days with the background, Naked liposomes, subtracted. The P values were measured using paired Student's t-tests.

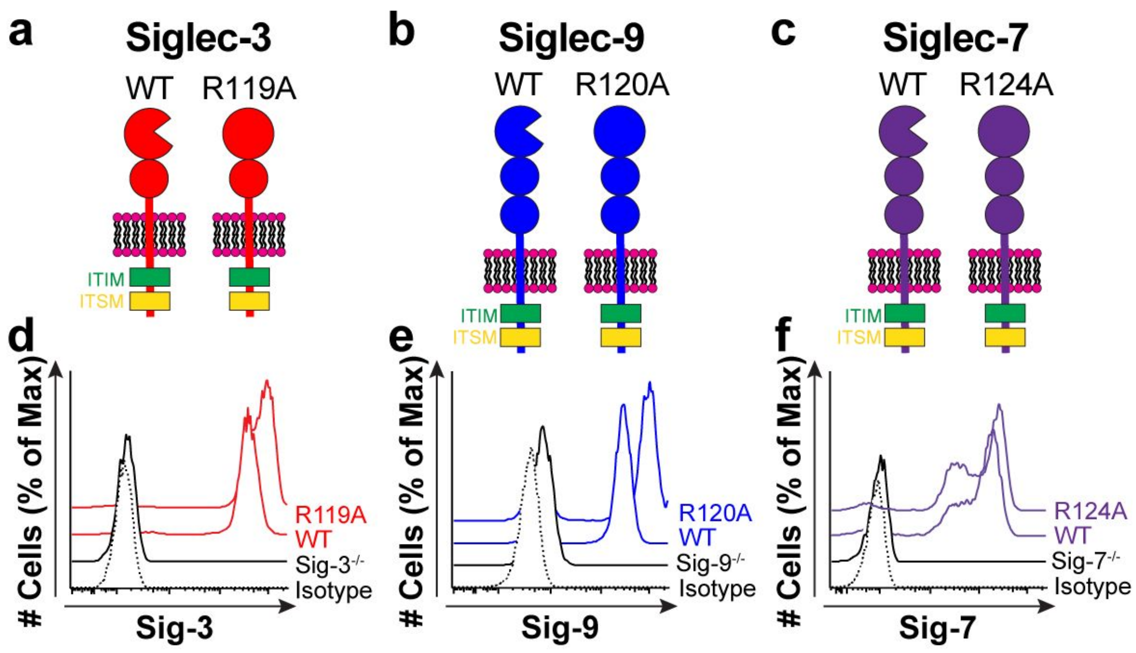

Figure S9. Creation of cells expressing Siglecs with the critical arginine residue required for binding mutated. (a,b,c) Cartoon depiction of WT Siglec-3 and R119A Siglec-3 (a), WT Siglec-9 and R120A Siglec-9 (b), and WT Siglec-7 and R124A Siglec-7 (c). (d,e,f) Expression levels of Siglec-3 (d), Siglec-9 (e), and Siglec-7 (f) in U937 WT cells virally transduced with empty vector (Sig$^{-/-}$), WT, or the critical arginine residue mutant as determined by flow cytometry.

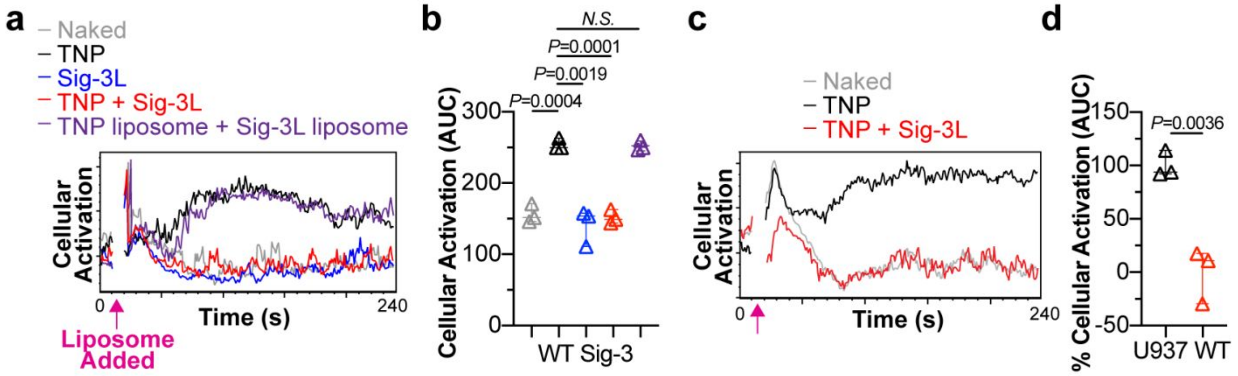

Figure S10. Calcium flux of Siglec-3 and FcγR engagement on separate liposomes or WT U937 cells. (a) Representative calcium flux curves of U937 WT Siglec-3 cells loaded with INDO-1-Am followed by anti-IgG2c antibody then stimulated with the indicated liposomes. The pink arrow represents the addition of liposomes 10 seconds after acquisition on the flow cytometer. (b) The amount of cellular activation (AUC) from the calcium curves quantified and plotted. (c,d) Representative flow cytometry calcium flux curves (c) and quantified area under the curves (d) of WT U937 cells administered the indicated liposomes. P values were quantified using unpaired Student's t-test and error bars are represented by median with 95% CI for three technical replicates.

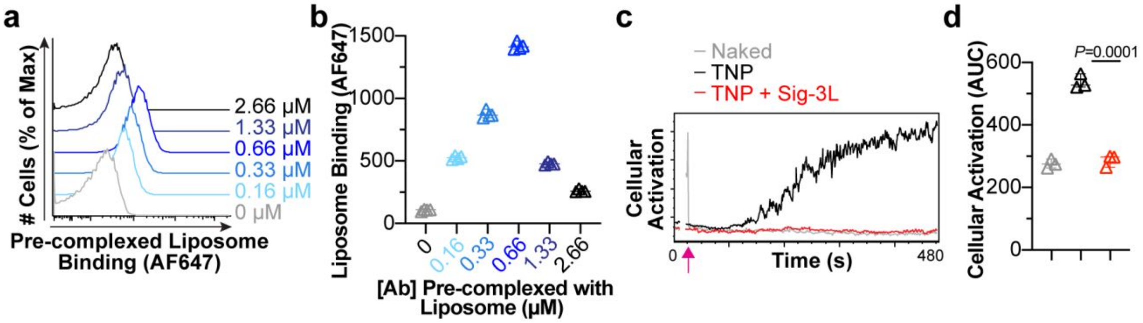

Figure S11. Liposomes pre-complexed with anti-TNP antibody bind and stimulate U937 cells. (a,b) Flow cytometry histogram (a) and plotted MFI values (b) of AF647 and TNP-containing liposomes pre-complexed with 0, 0.16, 0.33, 0.66, 1.33, or 2.66 μM of anti-TNP-IgG2c, (c,d) Representative calcium flux curves (c) and quantified area under the curve (d) of U937 WT cells stimulated with the indicated liposomes pre-complexed with 0.66 μM of antibody. The pink arrow represents the addition of liposomes 10 seconds after acquisition on the flow cytometer. *P* values were quantified using unpaired Student's t-test and error bars are represented by median with 95% CI for three technical replicates.

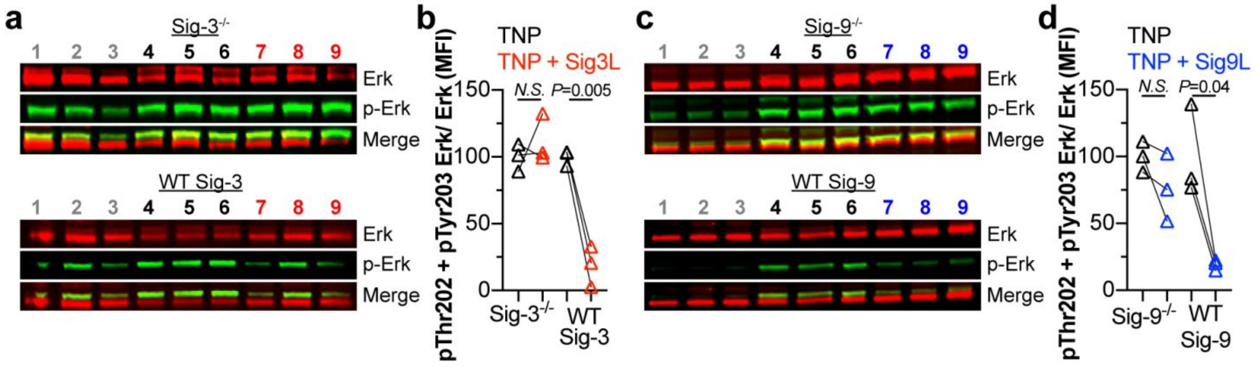

Figure S12. Phosphorylation of downstream protein Erk is decreased when Siglecs-3 or -9 are co-engaged with FcγRs. (a) Western blot of Erk and phosphorylated Erk (phosphoThreonine202 and phosphoTyrosine204) in Siglec-3$^{-/-}$ and WT Siglec-3 cells administered Naked liposomes (lanes 1-3), TNP liposomes (lanes 4-6), or TNP and 1% Sig-L liposomes (lanes 7-9). (b) MFI quantification of western blots plotting the ratio of pThr202 and pTyr202 Erk fluorescent signal over Erk fluorescent signal. (c) Siglec-9$^{-/-}$ and WT Siglec-9 cells stimulated with the indicated liposomes (Naked: lanes 1-3, TNP: lanes 4-6), or TNP + Sig-9L: 7-9) ran on a Western blot. Erk (red) and phosphorylated Erk (green) are stained. (d) MFI quantification of western blots plotting the ratio of pThr202 and pTyr202 Erk fluorescent signal over Erk fluorescent signal. ImageStudio Lite Software was used to visualize and quantify western blot data in b and d. P values in b and d were calculated using an unpaired Student's t-test in which three technical replicates were analyzed.

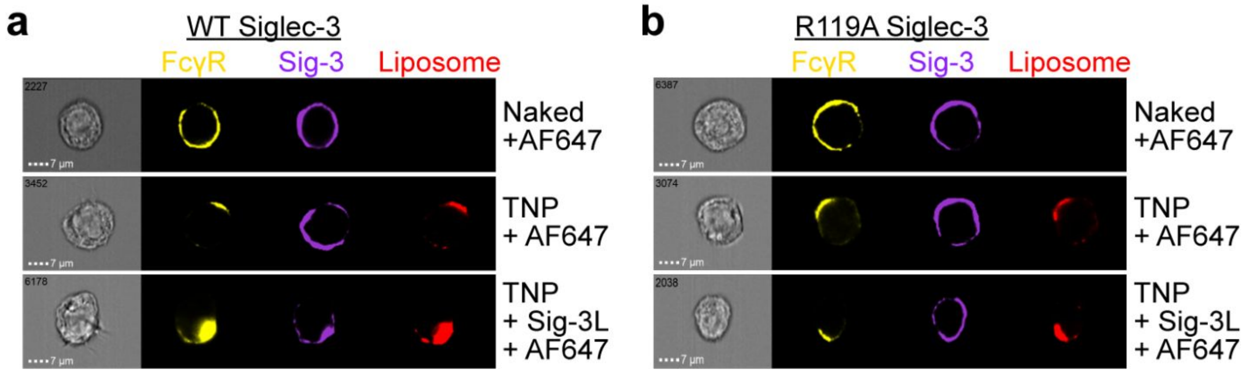

Figure S13. Imaging flow cytometry examining liposome, FcγR, and Siglec-3 colocalization. (a,b) Representative imaging flow cytometry data investigating the co-localization of FcγRI, Siglec-3, and liposomes after WT Siglec-3 cells (a) or R119A Siglec-3 cells (b) are given the indicated liposomes.

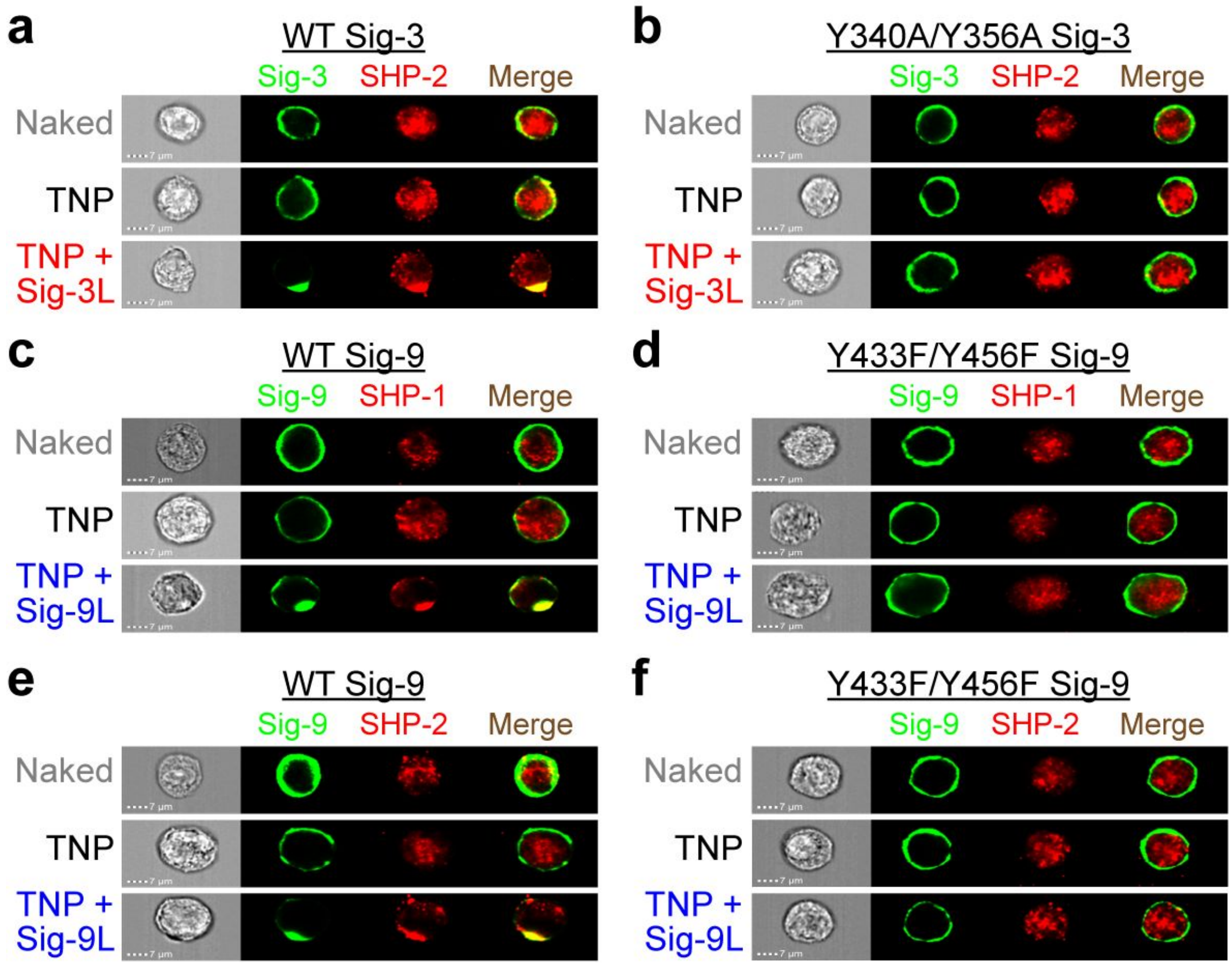

Figure S14. SHP-2 recruitment to Siglec-3 and -9. (a,b) Imaging flow cytometry representative image of SHP-2 recruitment to WT Siglec-3 (a) and Y340A/Y356A Siglec-3 (b) after administration of the indicated liposomes. (c,d) SHP-1 recruitment to WT Siglec-9 (c) and Y433F/Y456F Siglec-9 (d) after Naked, TNP, or TNP + Sig-9L liposome stimulation as visualized by imaging flow cytometry. (e,f) Representative imaging flow cytometry images showing SHP-2 recruitment to WT Siglec-9 (e) and Y433/Y456F Siglec-9 (f) when stimulated by Naked, TNP, or TNP + Sig-9L liposomes.

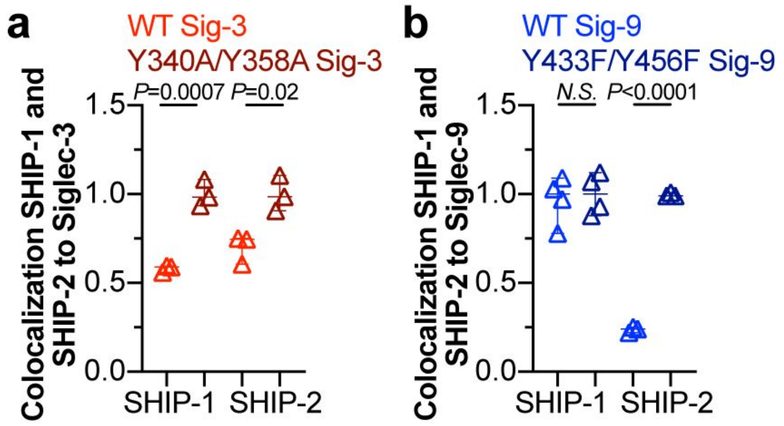

Figure S15. SHIP-1 and SHIP-2 recruitment to Siglec-3 and -9 by imaging flow cytometry. (a) Colocalization of SHIP-1 or SHIP-2 to Siglec-3 when WT Siglec-3 or Y340A/Y358A Siglec-3 cells are stimulated with TNP + Sig-3L liposomes. The values are normalized to the amount of colocalization in Y340A/Y358A Siglec-3 cells. (b) SHIP-1 and SHIP-2 colocalization to Siglec-9 in WT Siglec-9 or Y433F/Y456F Siglec-9 cells stimulated with TNP + Sig-9L liposomes. Plotted values are normalized to colocalization to Y433F/Y456F Siglec-9 cells. In a and b, the P values were calculated using unpaired Student's t-test and the data is presented as median with 95% CI for three (a) or four (b) technical replicates.

ITIM **ITSM**

Siglec-3---LHYASL-(X_{12})-TEYSEV
Siglec-5---LHYASL-(X_{18})-TEYSEI
Siglec-7---IQYAPL-(X_{17})-NEYSEI
Siglec-9---LQYASL-(X_{17})-TEYSEI

Figure S16. Sequencing alignment of Siglec-3, -5, -7, and -9 cytosolic tails. Immunomodulatory tyrosine-based inhibitory motif (ITIM) sequence and immunomodulatory tyrosine-based switch motif (ITSM) sequence of Siglecs-3, -5, -7, and -9. Single letter code for amino acids is used with an X representing any amino acid.

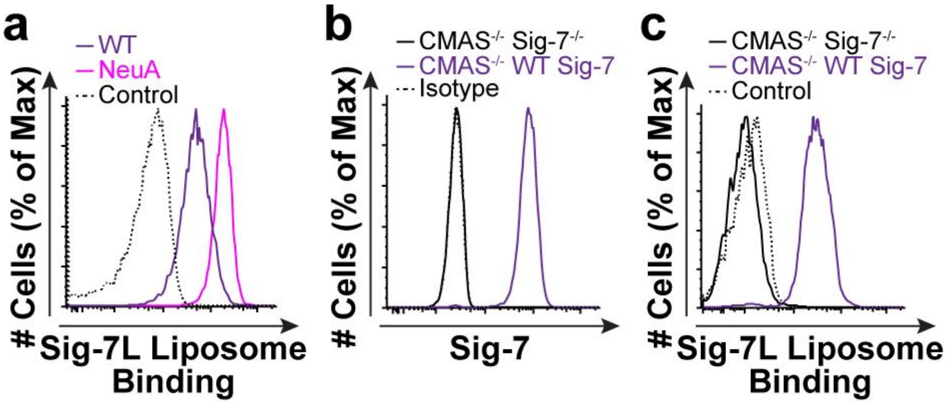

Figure S17. Removal of sialic acid from the cell surface increases Sig-7L liposome binding. (a) Flow cytometry of Sig-7L-PEG-DSPE and/or AF647-PEG-DSPE liposomes binding to U937 cells with or without Neuraminidase A digestion. (b,c) Siglec-7 expression (b) and Sig-7L-PEG-DSPE and AF647-PEG-DSPE liposome binding (c) to CMAS$^{-/-}$ Sig-7$^{-/-}$ cells virally transduced with an empty vector or WT Siglec-7 as measured using a flow cytometer.

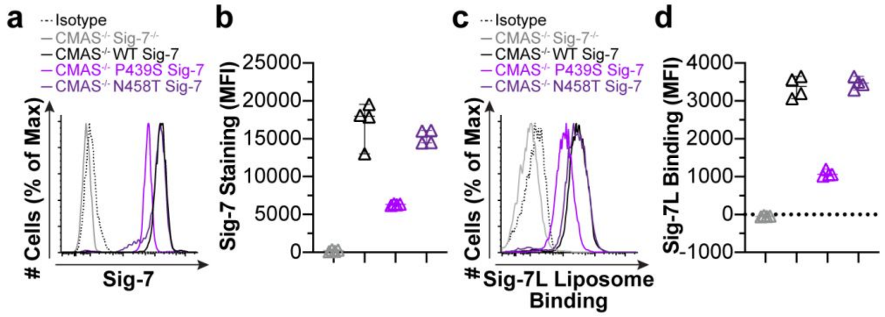

Figure S18. Creation of cells expressing Siglec-7 cytosolic mutants. (a,b) Four technical replicates of Siglec-7 antibody staining (a) and Sig-7L-PEG-DSPE and AF647-PEG-DSPE liposome binding (b) to CMAS$^{-/-}$ Sig-7$^{-/-}$ cells virally transduced with empty vector (CMAS$^{-/-}$ Sig-7$^{-/-}$), WT Siglec-7, P439S Siglec-7, and N458T Siglec-7.

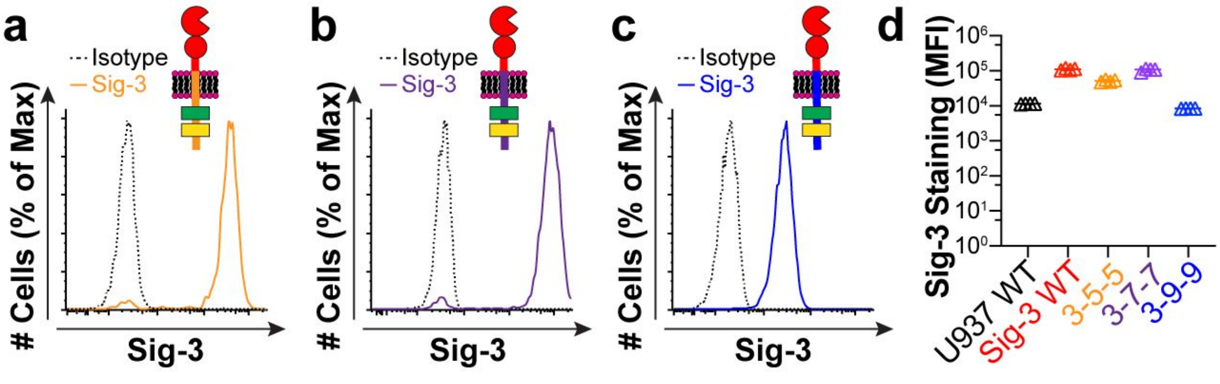

Figure S19. Creation of Siglec-3 chimera proteins. (a,b,c) Flow cytometry staining of chimeras 3-5-5 (a), 3-7-7 (b), and 3-9-9 (c). (d) Mean fluorescence intensity (MFI) values plotted of anti-Siglec-3 antibody staining by flow cytometry. Plotted values are four technical replicates, have isotype control MFI values subtracted, and are presented as median with 95% CI.

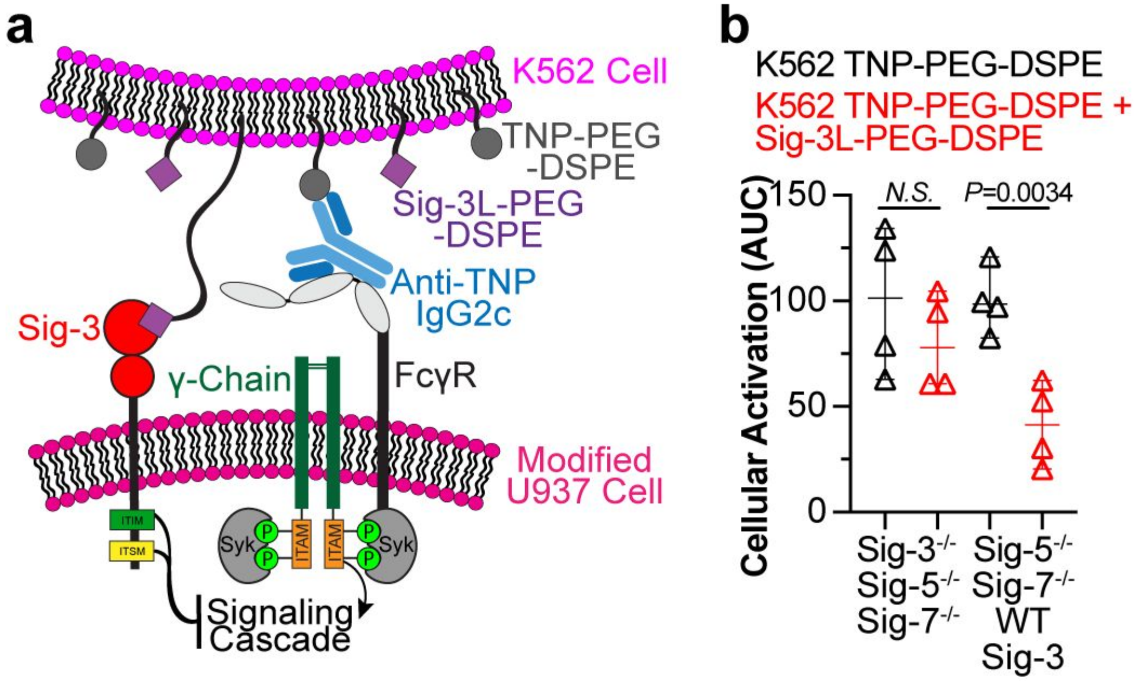

Figure S20. Lipid insertion of TNP-PEG-DSPE and/or Sig-3L-PEG-DSPE into K562 cells. (a) Schematic of lipid insertion into K562 Cells. (b) AUC from calcium flux of Siglec-3$^{-/-}$ or WT Siglec-3 cells given the indicated stimulating cells. The AUC values are with Naked cells area subtracted and normalized to the amount of activation from TNP-PEG-DSPE loaded cells. P values were obtained using unpaired Student's t-tests on four technical replicates and error bars are represented as median with 95% CI.

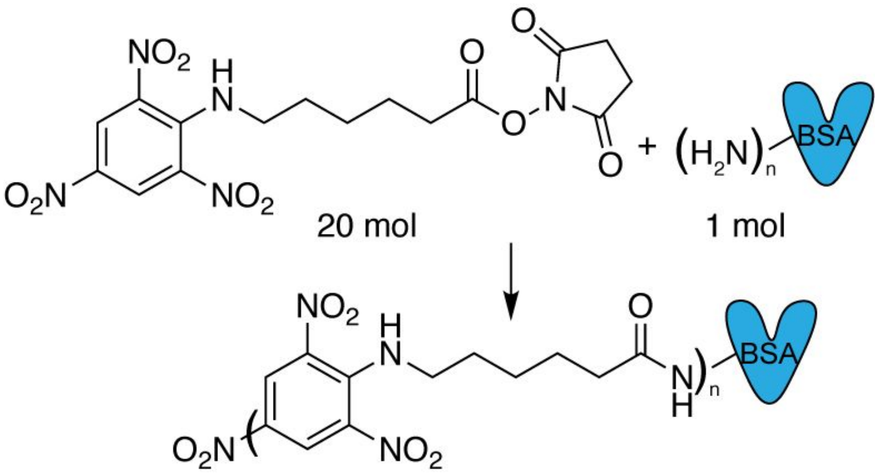

Figure S21. Trinitrophenyl conjugation to Bovine Serum Albumin. TNP-ε-aminocaproyl-OSu was linked to bovine serum albumin (BSA) using a 1:20 ratio of TNP-ε-aminocaproyl-OSu (in Dimethylformamide) to BSA (0.1 M $NaHCO_3$).

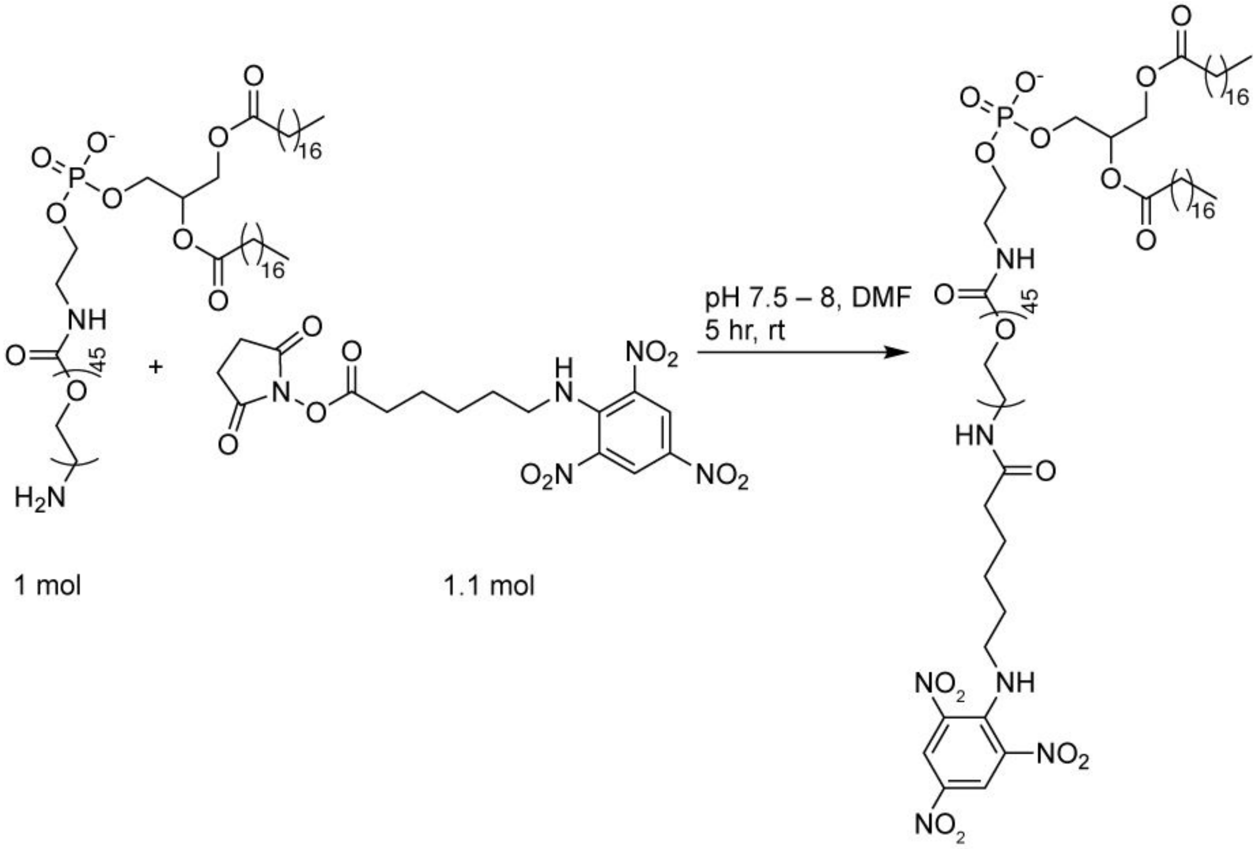

Figure S22. TNP-NHS conjugation to NH_2-PEG$_{(45)}$-DSPE. Conjugation of NHS-trinitrophenyl to NH_2-PEG$_{(45)}$-DSPE was done through combining 1 equivalent of NH_2-PEG$_{45}$-DSPE with 1.1 equivalent of NHS-trinitrophenyl in dried dimethylformamide (DMF) at a pH between 7.5 and 8 for 5 hr at room temperature. *N*,*N*-Diisopropylethylamine (DIPEA) was used to alter pH. After incubation, the DMF was removed using rotary evaporation.

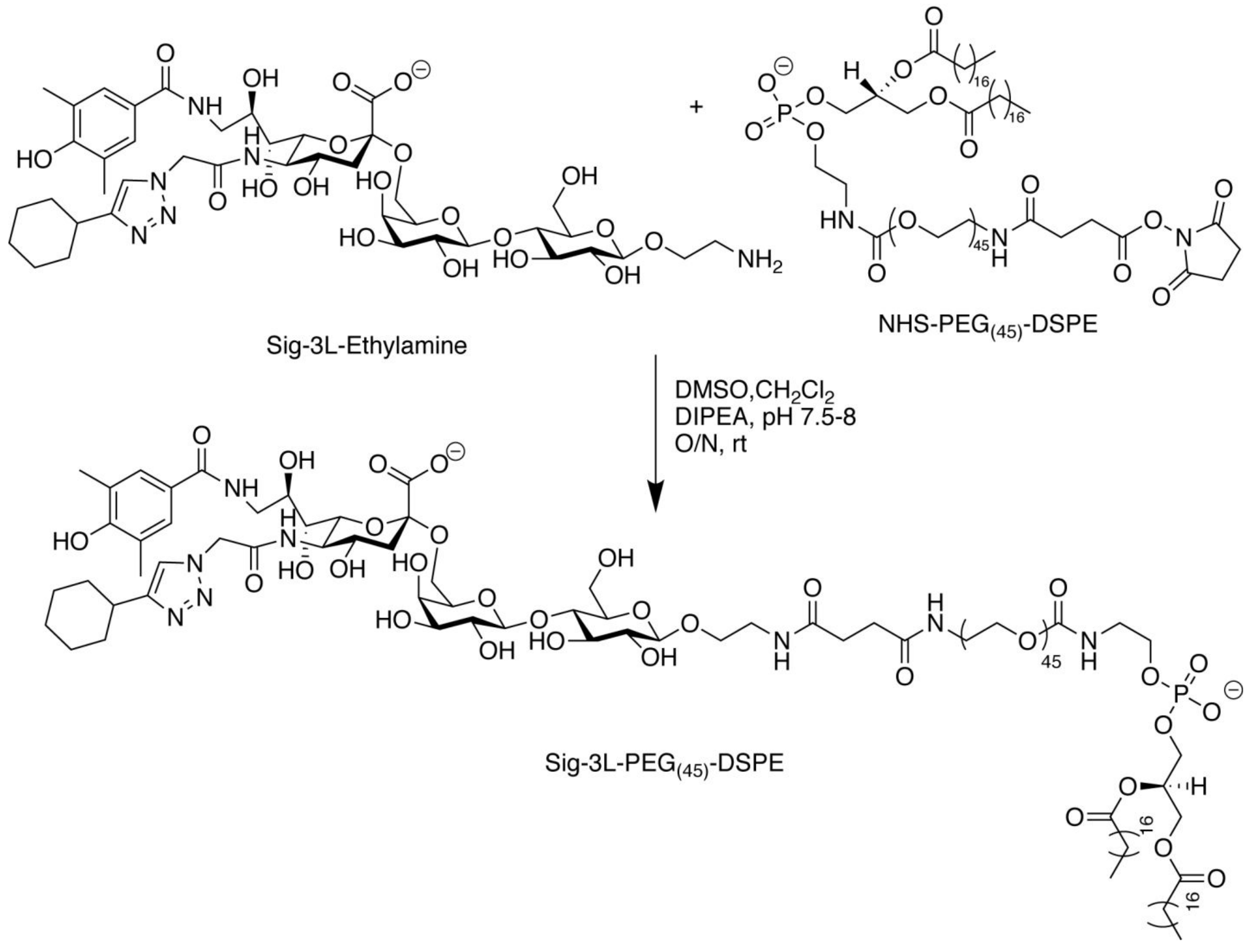

Figure S23. Siglec-3 ligand with an ethylamine conjugation to NHS-PEG$_{(45)}$-DSPE. Siglec-3 ligand harnessing an ethylamine was added to NHS-PEG$_{(45)}$-DSPE in a 1:1 mixture of dimethyl sulfoxide (DMSO) to dichloromethane (CH_2Cl_2). DIPEA was used to yield a pH between 7.5 and 8. The reaction mixture was left at room temperature overnight after which time the CH_2CL_2 and DMSO were removed via rotary evaporation and lyophilization, respectively.

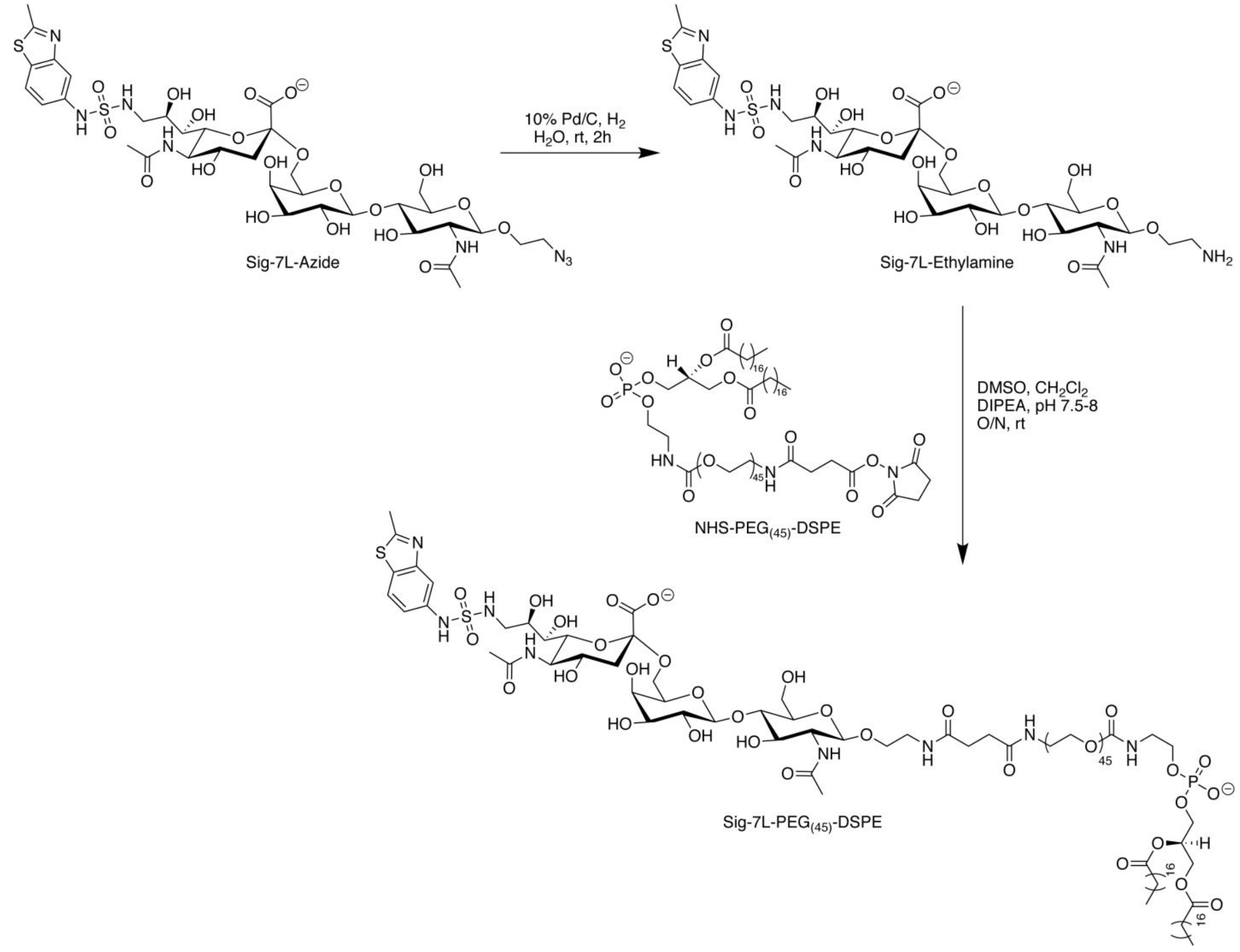

Figure S24. Siglec-7 ligand with an ethylamine conjugation to NHS-PEG$_{(45)}$-DSPE. Siglec-7 ligand containing an ethylazide aglycone (developed in a parallel manuscript in preparation) was first reduced to give the ethylamine format, followed by reaction with NH_2-PEG$_{(45)}$-DSPE in a 1:1 mixture of dimethyl sulfoxide (DMSO) to dichloromethane (CH_2Cl_2). DIPEA was used to yield a pH between 7.5 and 8. The reaction mixture was left at room temperature overnight then the CH_2CL_2 and DMSO were removed via rotary evaporation and lyophilization, respectively

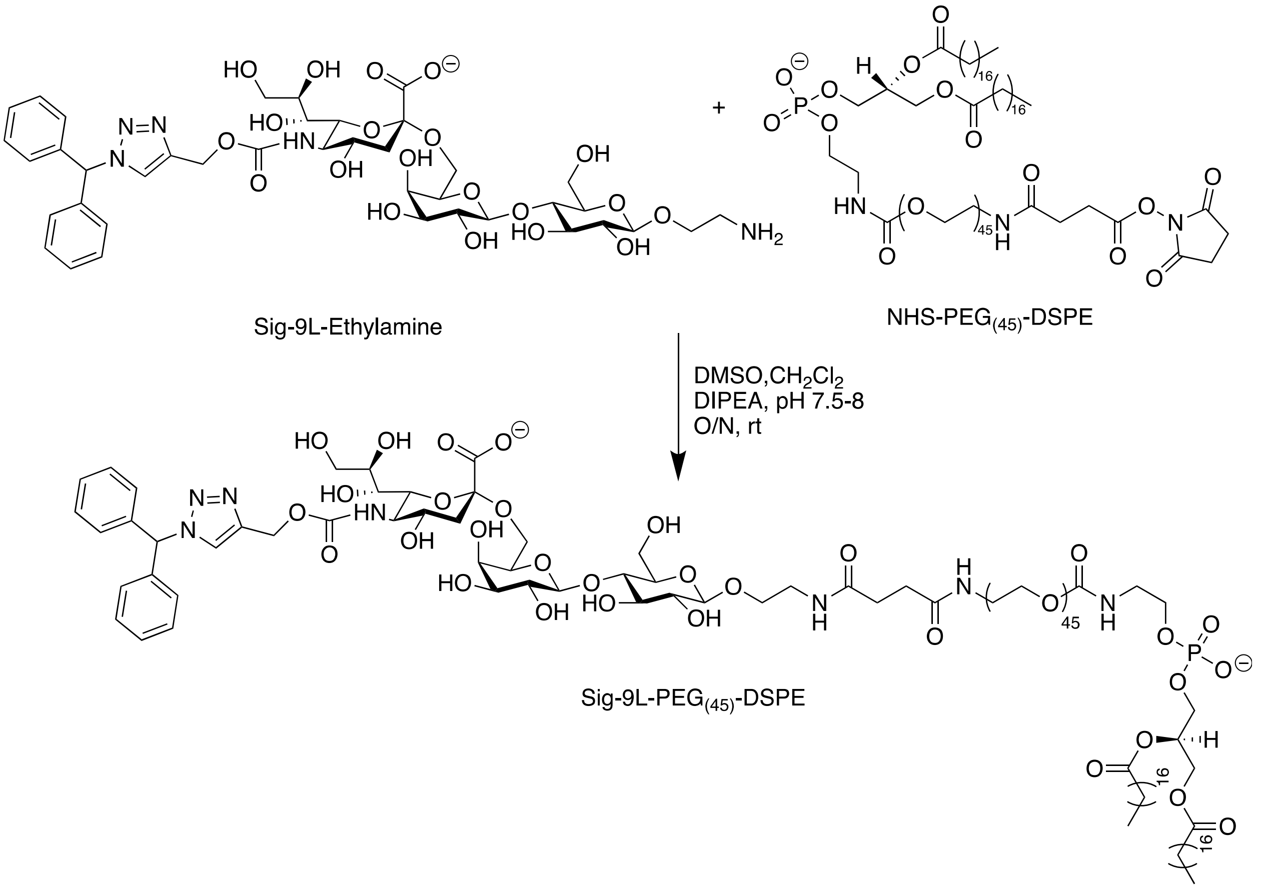

Figure S25. Siglec-9 ligand with an ethylamine conjugation to NHS-PEG$_{(45)}$-DSPE. Ethylamine-Siglec-9 ligand and NHS-PEG$_{(45)}$-DSPE were mixed together a 1:1 mixture of DMSO to CH_2Cl_2 at a pH between 7.5 and 8 (DIPEA) and left overnight. After incubation, the CH_2Cl_2 was removed using rotary evaporation followed by removal of DMSO via lyophilization.
