# Supplementary material for: Dissecting the Ability of Siglecs To Antagonize Fcγ Receptors
Source: ACS Cent Sci. 2024 Jan 17;10(2):315–30. doi: 10.1021/acscentsci.3c00969 (PMC10906256; doi:10.1021/acscentsci.3c00969)
Supplement: Supplementary file 2 — oc3c00969_si_002.pdf [file oc3c00969_si_002.pdf]

oc-2023-00969u.R1

Name: Peer Review Information for "Dissecting the ability of Siglecs to antagonize Fcg receptors"

First Round of Reviewer Comments

Reviewer: 1

Comments to the Author

Fc receptors are key mediators of antibody effector functions, and detailed information about the mechanisms of regulation is useful. In this study, the authors carry out an in depth and careful analysis of the effects of simultaneously engaging Siglecs and Fc receptors. The authors use liposomes that can interact with an Fc receptor and one of Siglec 3, 5, 7, or 9 as tools to evaluate the effects of dual engagement. They found that Siglec 3 and Siglec 9 can fully inhibit FcgammaR activation, an effect involving ITIM and ITSM motifs. SHP-1 recruitment was ITIM dependent while SHP-2 recruitment was ITSM dependent. The results from this liposomal system support prior reports that Siglecs modulate Fc receptors in vivo and elucidate potential mechanisms by which modulation occurs.

Overall, it's a very nice study. The paper includes extensive data from numerous experiments. A great set of controls is used, and the work is rigorous. In addition, the manuscript is well written. However, I'm not sure it will appeal to the broad chemistry-centric readership of ACS Central Science. The study seems better suited for a journal like the Journal of Biological Chemistry.

I've included some suggestions for revisions below:

Throughout the methods section, there are some details that are missing.

For the mouse studies, the authors should list the sexes of the animals used in the study.

In the section on production of TNP IgG- the authors should specify the volumes of injections. Did the boosts have adjuvant (e.g. IFA)? Any data for purity of antibodies? Any characterization of specificity of those antibodies?

For the antibody binding assay section - which exact secondary antibodies were used (list full name and catalog number)?

How much neuraminidase A was used and what was the source? What was the volume of the reaction?

The authors should include the volume and concentrations of the TNP-BSA conjugation reaction.

For the lipid insertion steps – what were the volumes?

For the TNP staining – what was the concentration of the starting antibody (which was dilution 1:200)? What volumes were used for various steps?

In the figure legends, the authors should add the number of replicates, especially when there are error bars.

Reviewer: 2

Comments to the Author

McCord and colleagues report on the role of inhibitory Siglec receptors in modulating the signaling of Fc gamma receptors. The authors used liposomes decorated with ligands for the different Siglec receptors. As a model, McCord and colleagues manipulated U937 cells to express specific Siglec receptors and used IgG against TNP that was together with Siglec ligands presented on liposomes. The authors found that co-engagement of Siglec-3 and Siglec-9 together with TNP inhibited activation of U937 cells. They further demonstrate that SHP1 and SHP2 recruitment as downstream signaling is regulated differently by ITIM and ITSM. Furthermore, the authors investigated the role of Siglec-mediated inhibition in cell-cell interactions. This is a very well planned, and controlled study. The following suggestions could be taken into consideration before publication of this work.

1. How is the effect of the liposomes used on primary cells? Could that also be used to test the effects on primary NK cells (e.g. Siglec-7/-9 signaling)?

2. In this context: is there any speculation why Siglec-7 is not completely inhibiting calcium flux in U937 cells activated via IgG?

3. The approach to use chimeric Siglec receptors to investigate Siglec-5 is interesting. It would be interesting to analyze the role the paired Siglec-14 receptor in parallel with the same approach.

4. Calcium flux was used as a main readout. Would it be also interesting into looking into surface expression of markers upon liposomal incubation?

5. What is the effect of CMAS depletion on cell growth, viability etc?

Reviewer: 3

Comments to the Author

This is an impressive and ambitious study with excellent experimental design and thorough analysis. The authors have exploited novel tools to dissect the ability of selected Siglecs to inhibit FcR-mediated cellular activation. The data are clear and well presented. I have generated a PDF file with specific comments for the attention of the editor and authors.

My main issues relate to the lack of results from more physiologically relevant models. For instance, I believe that pre-incubation of the liposomes with the antibodies rather adding the antibodies to the cells and then washing before incubating with liposomes, would be closer to the situation in the host.

Also I am not sure how the expression levels of the lentivirus-encoded Siglecs compare to their endogenous levels and if the effect of the endogenous Siglec receptors were tested. Did I miss it? For instance, as far as I can tell the authors should have access to Siglec5-/-, Siglec9-/- U937 cells and Siglec3-/-, Siglec5-/-, Siglec9-/- U937 cells. Do these cells respond differently to liposomes displaying TNP and Siglec3L?

Finally, emphasis is placed on how tumour cells can used enhanced sialylation to inhibit FcR-mediated effects. I wonder if the authors could screen tumour cells for high and low sialylation, load them with TNP and test how they affect FcR function?

Author's Response to Peer Review Comments:

Reply to Reviewers

Reviewer: 1

Fc receptors are key mediators of antibody effector functions, and detailed information about the mechanisms of regulation is useful. In this study, the authors carry out an in depth and careful analysis of the effects of simultaneously engaging Siglecs and Fc receptors. The authors use liposomes that can interact with an Fc receptor and one of Siglec 3, 5, 7, or 9 as tools to evaluate the effects of dual engagement. They found that Siglec 3 and Siglec 9 can fully inhibit FcgammaR activation, an effect involving ITIM and ITSM motifs. SHP-1 recruitment was ITIM dependent while SHP-2 recruitment was ITSM dependent. The results from this liposomal system support prior reports that Siglecs modulate Fc receptors in vivo and elucidate potential mechanisms by which modulation occurs.

Overall, it's a very nice study. The paper includes extensive data from numerous experiments. A great set of controls is used, and the work is rigorous. In addition, the manuscript is well written. However, I'm not sure it will appeal to the broad chemistry-centric readership of ACS Central Science. The study seems better suited for a journal like the Journal of Biological Chemistry.

Reply: Thank you for the positive assessment of our work. We strongly believe that the chemical biology approach we have leveraged here to study the interactions between two opposing immunomodulatory receptors is of great interest to a broad audience. We have added the following text to the conclusions of our revised manuscript to make this point more clear:

"Although this study focused on the ability of Siglecs to antagonize the specific family of FcγRs, this approach should find more widespread use for systematically studying the regulation of one immunomodulatory receptor by another."

I've included some suggestions for revisions below:

1. Throughout the methods section, there are some details that are missing.

Reply: Thank you for the feedback. We have gone through our methods carefully and made numerous corrections and additions.

2. For the mouse studies, the authors should list the sexes of the animals used in the study.

Reply: Information on the sex of the mice was added to the methods:

The manuscript was changed from:

"C57Bl/6J mice were obtained from Jackson Laboratory and bred at The Scripps Research Institute (TSRI). Animal studies were approved by the TSRI Institutional Animal Care and Use Committee."

To:

"Female C57Bl/6J mice were obtained from Jackson Laboratory and bred at The Scripps Research Institute (TSRI). Animal studies were approved by the TSRI Institutional Animal Care and Use Committee."

3. In the section on production of TNP IgG- the authors should specify the volumes of injections. Did the boosts have adjuvant (e.g. IFA)? Any data for purity of antibodies? Any characterization of specificity of those antibodies?

Reply: The volumes of injections were added to our methods section. No adjuvant was given for secondary boosts to increase high affinity clones. The resulting antibodies were subjected to ELISA for characterization and purified by Protein G chromatography.

The method section in our manuscript was changed from:

"C57BL/6J mice were immunized intraperitoneally with 20 μg TNP:KLH (15:1) emulsified in Imject incomplete Freund's adjuvant (Thermo Scientific). At four and 10 weeks, animals were boosted intraperitoneally with 5 μg in PBS,"

To:

"Female C57BL/6J mice were immunized intraperitoneally with 20 μg TNP:KLH (15:1) emulsified in 100 μL of Imject incomplete Freund's adjuvant (Thermo Scientific). At four and 10 weeks, animals were boosted intraperitoneally with 5 μg in 100 μL of PBS,"

4. For the antibody binding assay section - which exact secondary antibodies were used (list full name and catalog number)?

Reply: We have addressed this question with the addition of a SI Table (Table S3) including exact antibodies, clones, catalogue number, and company for each antibody used. Under the *Siglec expression of neutrophils from whole blood* and *antibody binding assays* sections of our methods, we wrote:

"A more detailed list of antibodies used can be found in Table S3."

And:

"Please see Table S3 for the details regarding the clones and catalogue number of each antibody used."

5. How much neuraminidase A was used and what was the source? What was the volume of the reaction?

Reply: This information was added to our method section. Specifically, we have changed:

"About two million cells were resuspended in PBS with or without Neuraminidase A and shaken at 37 ºC for 1 hr."

To:

"About two million cells were resuspended in 1000 μL of PBS with or without 50 μL of 0.3 mg/mL Neuraminidase A from *Arthrobacter ureafaciens* and shaken at 37 ºC for 1 hr."

6. The authors should include the volume and concentrations of the TNP-BSA conjugation reaction.

Reply: The precise volumes and concentrations for this reaction were added to the methods resulting in the manuscript. The text has been changed from:

"Linking TNP-e-aminocaproyl-OSu to BSA. TNP-e-aminocaproyl-OSu was linked to bovine serum albumin (BSA) using a 1:20 ratio of TNP-e-aminocaproyl-OSu (in Dimethylformamide) to BSA (0.1 M $NaHCO_3$)."

To:

"TNP-e-aminocaproyl-OSu was linked to bovine serum albumin (BSA) using a 1:20 ratio of 0.1 M TNP-e-aminocaproyl-OSu (in 68.6 µL Dimethylformamide) to 0.1 M BSA (in 500 µL $NaHCO_3$)."

7. For the lipid insertion steps – what were the volumes?

Reply: The final volume and concentration of the lipid insertion has been added to the methods changing:

"10 µM of TNP-PEG-DSPE and/or Sig-3L-PEG-DSPE in PBS were then added to cells and left to passively diffuse into the cell membrane through incubation at 37 ºC for 2 hr."

To:

"10 µM of TNP-PEG-DSPE and/or Sig-3L-PEG-DSPE were added to 500 µL of cells in PBS and left to passively diffuse into the cell membrane through incubation at 37 ºC for 2 hr."

8. For the TNP staining – what was the concentration of the starting antibody (which was dilution 1:200)? What volumes were used for various steps?

Reply: The concentration of the initial antibody has been added to the methods. The volumes were scaled depending on the number of samples tested each day. The overall dilutions are provided so that readers can scale further reactions. The text was changed from:

"A mouse anti-TNP-IgG2b antibody was added to half of the cell pellets in a 1:200 ratio of antibody:FACS buffer and the cells were incubated at 4 ºC for 25 min."

To:

"A 2.5 mg/mL solution of mouse anti-TNP-IgG2b antibody was added to half of the cell pellets in a 1:200 ratio of antibody:FACS buffer and the cells were incubated at 4 ºC for 25 min."

9. In the figure legends, the authors should add the number of replicates, especially when there are error bars.

Reply: Thank you for this suggestion. We have gone through our figure legends and included the number of replicates for scatter dot plots that we had not previously specified.

Reviewer: 2

McCord and colleagues report on the role of inhibitory Siglec receptors in modulating the signaling of Fc gamma receptors. The authors used liposomes decorated with ligands for the different Siglec receptors. As a model, McCord and colleagues manipulated U937 cells to express specific

Siglec receptors and used IgG against TNP that was together with Siglec ligands presented on liposomes. The authors found that co-engagement of Siglec-3 and Siglec-9 together with TNP inhibited activation of U937 cells. They further demonstrate that SHP1 and SHP2 recruitment as downstream signaling is regulated differently by ITIM and ITSM. Furthermore, the authors investigated the role of Siglec-mediated inhibition in cell-cell interactions. This is a very well planned, and controlled study. The following suggestions could be taken into consideration before publication of this work.

1.How is the effect of the liposomes used on primary cells? Could that also be used to test the effects on primary NK cells (e.g. Siglec-7/-9 signaling)?

Reply: This is a good suggestion. Upon further review of the literature, NK cells primarily express FcγRIII. In our original manuscript, we had stated that NK cells also express FcγRII, which is only partially correct because it is only in a subset of patients with a polymorphism (PMID: 9516136). Therefore, we have fixed this statement in our revised discussion. Accordingly, testing NK cells would specifically examine the ability of Siglec-7 and -9 to negatively regulate FcγRIII, which would be difficult to compare with the rest of the results presented in our manuscript that predominantly reflect activation of cells through FcγRI with a small contribution from FcγRII. Therefore, while we think this is a really interesting question, we feel it is beyond the scope of our manuscript to test finding in another cell type with a different receptor. Our manuscript was changed from:

"As FcγRII and FcγRIII are expressed on NK cells and can activate them upon being crosslinked,[68,69] it will be of interest to investigate the ability of Siglec-7 and -9 to more broadly inhibit FcRs on NK cells.

To

"As NK cells primarily express FcγRIII, which can also be activated upon crosslinking,[71,72] it will be of interest to investigate the ability of Siglec-7 and -9 to more broadly inhibit FcγRIII on NK cells."

2.In this context: is there any speculation why Siglec-7 is not completely inhibiting calcium flux in U937 cells activated via IgG?

Reply: Our results depict that the non-consensus proline in the ITIM of Siglec-7 or the asparagine in the ITIM-like motif of Siglec-7 results in differences in inhibition. We are able to conclude this because when upon mutation of these residues to that of amino acids present in the ITIM and ITSM of Siglec-3, -5, and -7, the ability of Siglec-7 to inhibit the responses through FcγR was dramatically enhanced.

3.The approach to use chimeric Siglec receptors to investigate Siglec-5 is interesting. It would be interesting to analyze the role the paired Siglec-14 receptor in parallel with the same approach.

Reply: We agree! However, without a selective ligand to engage Siglec-5/14, which share an identical glycan binding specificity, it would require creating a chimeric construct with the extracellular domains of Siglec-3 and the transmembrane and cytoplasmic tail of Siglec-14. Cloning such a construct and stable transfection in cells would take a minimum of two months, which is significantly longer than we were afforded for the revisions. Therefore, while we think this is a really interesting question to address in the future, we feel it is beyond the scope of our current

manuscript. We would like to point out that a similar approach - of turning an inhibitory Siglec into an activatory Siglec - was taken recently for Siglec-3, where the transmembrane segment and cytoplasmic (ITAM-containing) tail of DAP12 was stitched on (PMID: 34188106).

4.Calcium flux was used as a main readout. Would it be also interesting into looking into surface expression of markers upon liposomal incubation?

<u>Reply</u>: We agree that it would be interesting to evaluate additional readouts of activation. We wish to point out that our manuscript investigated several readouts of cellular activation in addition to Calcium flux, including activation of downstream signaling components Akt and Erk, and all of our readouts of cellular activation provide consistent results on the ability of Siglecs to inhibit FcγR.

5.What is the effect of CMAS depletion on cell growth, viability etc?

<u>Reply</u>: The essential role of sialic acid to living systems is a physiological phenomenon, where it is crucial *in vivo*. However, in standard cultured cell lines, removal of sialic acid biosynthesis is routinely performed by many labs without any significant effects on cellular viability. Still, we agree that this is an important point to address and, therefore, have assessed the growth rates of WT vs CMAS$^{-/-}$ U937 cells. The results are presented below for review only, demonstrating similar growth rates between the two cells.

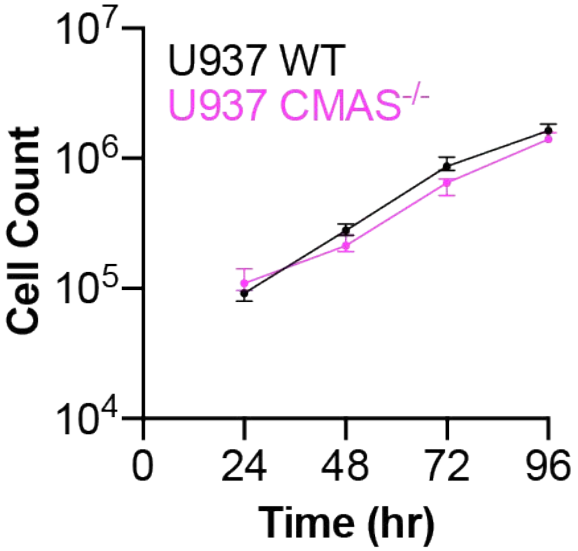

Figure for Review: Growth rates of U937 WT and U937 CMAS-/- cells. Three replicates of U937 WT cells (black) and U937 CMAS-/- cells (pink) were counted every 24 hr for 4 days. The error bars are presented as median with 95% confidence interval.

Reviewer: 3

This is an impressive and ambitious study with excellent experimental design and thorough analysis. The authors have exploited novel tools to dissect the ability of selected Siglecs to inhibit FcR-mediated cellular activation. The data are clear and well presented. I have generated a PDF file with specific comments for the attention of the editor and authors.

Reply: Thank you for the positive assessment of our manuscript and all of the feedback to help improve it.

My main issues relate to the lack of results from more physiologically relevant models. For instance, I believe that pre-incubation of the liposomes with the antibodies rather adding the antibodies to the cells and then washing before incubating with liposomes, would be closer to the situation in the host.

Reply: We agree with the reviewer that this is a good point to check. Therefore, we have carried out a series of experiments using this approach. We first optimized pre-complexation of antibody with liposomes and examined binding (Fig. S11A,B). We then took optimized pre-complexing conditions and showed that they induce cellular activation (Fig. S11C). Importantly, pre-complexing with liposomes containing Siglec-3L did not activate cells, indicating that under pre-complexed conditions, Siglec-3 can also fully inhibit FcγRs.

"Liposomes pre-complexed with the anti-TNP-IgG2c also bound to U937 cells (Figure S11A,B). Excess anti-TNP-IgG2c outcompeted binding of pre-complexed anti-TNP-IgG2c on liposomes. Optimized pre-complexed conditions led to stimulation of U937 cells and anti-TNP-IgG2c precomplex with liposomes containing Sig-3L did not activate U937 cells (Figure S11C,D)."

Also I am not sure how the expression levels of the lentivirus-encoded Siglecs compare to their endogenous levels and if the effect of the endogenous Siglec receptors were tested. Did I miss it? For instance, as far as I can tell the authors should have access to Siglec5-/-, Siglec9-/- U937 cells and Siglec3-/-, Siglec5-/-, Siglec9-/- U937 cells. Do these cells respond differently to liposomes displaying TNP and Siglec3L?

Reply: The expression levels of lentivirus-encoded Siglecs are higher than endogenous levels, however full inhibition of FcγRs by Siglec-3 is still observed upon engaging endogenous Siglec-3. This is now illustrated in Supplementary Figure S10 of our revised manuscript. The reason lentivirus-encoded Siglec results were depicted in our manuscript was because it allowed us to investigate various mutants and all transduced mutants were expressed at relatively the same level. We have also added the following text to describe these results:

"and U937 WT cells expressing endogenous Siglec-3 also exhibited decreased FcγR activation when administered TNP + Sig-3L liposomes (**Figure S10C,D**)."

Finally, emphasis is placed on how tumour cells can used enhanced sialylation to inhibit FcR-mediated effects. I wonder if the authors could screen tumour cells for high and low sialylation, load them with TNP and test how they affect FcR function?

Reply: We agree with the reviewer that this would be an excellent line of experiments and something that we are very interested in pursuing in the future. To do so will require a careful examination of many cancer cell lines to look for ones that have high levels of ligands for Siglec-3 and/or -9 and then will ideally require the generation of a sialic acid-knockout in this line, which will take a significant amount of time. Further to this, Siglecs-3, -5, -7, and -9 have overlapping binding specificities (PMID: 34661385), so we could potentially be engaging multiple Siglecs at a time. The results in our manuscript with the cell-cell experiment serves as a proof-of-concept. In addition, we have added the following sentence to our manuscript to address this comment:

"In the future, it will be interesting to test this cell-cell based assay in tumor cells with high sialylation to better understand the ability of Siglecs to inhibit immune responses in the context of a natural immunological synapse between myeloid cells and cancerous cells."

Specific comments:

1. Page 4, Lines 46-47
"antibody-mediated neutrophil cytotoxicity against tumor cells can be improved by either removing sialic acid on the surface of tumor cells or using a Siglec-9 blocking antibody."
Authors should emphasise that this finding justifies their experimental approach.

Reply: Thank you for the suggestion. To further clarify that this justifies our experimental approach, we have added the sentence to our introduction:

"leading us to question whether Siglecs can directly inhibit FcγR-mediated activation of cells"

2. Page 5, Line 38
The authors should describe early on the FcR expressed by U937 cells.

Reply: We wish to kindly point out that FcγR expression in U937 cells was measured by flow cytometry early in our results (Figure 1C).

3. Lines 43-44
"their FcRs are free, unlike primary monocytes whose FcRs are saturated by IgG"
Please refer to the original study, not a review as this is an important consideration. I believe all assays are done in the presence of FCS, which should have reduced IgG levels. This should be pointed out.

Reply: Thank you for pointing this out. We have added in two more references (PMID: 20805361 and PMID: 23293080). To be more cautious with our terminology, we have changed the manuscript from:

"their FcγRs are free, unlike primary monocytes whose FcγRs are saturated by IgG[45],"

To:

"their FcγRs are relatively free due to reduced levels of IgG in culture media, unlike primary monocytes whose FcγRs are mostly saturated by IgG[45-47]"

4. Line 49
" lentiviral transduction"
is it worth describing which promoter drives expression of the different siglecs?

Reply: Thank you for the suggestion. We added in the following:

"U937 cell lines with or without each Siglec were developed by first disrupting expression of each Siglec by CRISPR/Cas9, followed by re-introduction of each Siglec through lentiviral transduction or an empty vector control under the EF1α promoter."

5. Lines 51-56
"These cell lines were used to test the ability of each Siglec to antagonize FcRs by using liposomes co-displaying a high affinity, Siglec-specific ligand along with a ligand to engage FcR."
Did you test if deletion of the different Siglecs in U937 cells use the WT and Siglec3 -/-, Sigl3c5 and Siglec7-/- affected the response of the cells to TNP Liposomes containing different Siglec ligands? This would be important to ensure that Siglecs expressed at physiological levels also have inhibitory effects.

Reply: We were able to test the ability of Siglec-3 on U937 cells to inhibit FcγRs as Siglec-3 is expressed endogenously. Siglec-9 is barely expressed on U937 cells, Siglec-7 is highly masked on U937 cells, and we do not have a ligand for engaging Siglec-5. Therefore, we were only able to test Siglec-3 inhibition at physiological levels. As we demonstrated above, endogenous Siglec-3 was capable of fully inhibiting FcγR activation when engaged by its ligand.

6. Page 6
Lines 5-6
"To engage FcRs, we developed a set of mouse monoclonal anti-trinitrophenyl (TNP) IgG antibodies of the IgG1, IgG2c, and IgG2b isotypes"
Is it suitable to use mouse IgGs when using a human cell line? This should be highlighted like a potential limitation. Also, these antibodies will be glycosylated and I wonder if this should be mentioned.

Reply: The binding of mouse IgG subclasses to human FcγR classes has been studied extensively in 2020 (PMID: 32947169). Mouse IgG2a/c is known to bind human FcγRs, and its glycosylation does not alter this binding. Thus, it was suitable to use in these studies.

7. Line 12-16
"U937 cells were pre-incubated with anti-TNP-IgG1, anti-TNP-IgG2c, or anti-TNP-IgG2b, washed, probed with fluorescent TNP-liposomes, and measured by flow cytometry"
Wouldn't it make more sense to preincubate the liposomes with the antibodies so they are presented to the cells in a polymeric fashion? Did you test if it makes a difference?

Reply: This is a good suggestion. We had started the project performing the assay this way but went away from it as we found that it required significantly more antibody to saturate the TNP molecules. However, when we found that antibodies could be incubated with cells, as we performed in the manuscript, we stuck with that approach. However, we agree with the reviewer that this is an interesting point and, therefore, pursued experiments along these lines to see if it makes a difference.

First, we optimized this approach by performing a titration of the anti-TNP IgG2c with the TNP liposome and tested these for their ability to bind and stimulate U937 cells. As shown in Fig. S11a,b of our revised manuscript, the optimal amount of antibody was determined that facilitated

binding to cells. Likewise, in Fig. S11c,d we see the same trends for the stimulation of cells. Excess amounts of antibody likely blocks binding of antibody pre-complexed on the liposomes.

8. Line 18
“in the highest liposome binding”
I would say that incubation with IgG1 and IgG2b did not induce any liposome binding

Reply: Thank you for your suggestion. We changed our manuscript from:

“Pre-incubation with anti-TNP-IgG2c resulted in the highest liposome binding (Figure 1B), which is in line with mouse IgG2a/c having the highest affinity for human FcγRI.[46]”

To:

“Pre-incubation with anti-TNP-IgG2c resulted in significant liposome binding, while pre-incubation with anti-TNP-IgG1 or anti-TNP-IgG2b resulted in little to no binding (Figure 1B). This is in line with mouse IgG2a/c having the highest affinity for human FcγRI,[48]”

9. Line 21:
“having the highest affinity for human FcRI”
refer to FcR expression in the cell line.

Reply: We have changed the manuscript from:

“which is in line with mouse IgG2a/c having the highest affinity for human FcγRI.”

To:

“which is in line with mouse IgG2a/c having the highest affinity for human FcγRI,[48] and FcγRI being highly expressed on U937 cells (Figure 1C).”

10. Lines 27-31
“TNP-liposome binding to cells incubated with anti-TNP-IgG2c was abrogated by prior treatment of cells with an Fc-blocking antibodies, indicating that the binding was FcR dependent”
Shouldn't it be "partially-dependent"? There is residual binding in the presence of Fc block.

Reply: We agree. Binding is not 100% abrogated, which may reflect Fc blocking not being perfect. We have made the following modification to our revised manuscript:

From:
“TNP-liposome binding to cells incubated with anti-TNP-IgG2c was abrogated by prior treatment of cells with an Fc-blocking antibodies, indicating that the binding was FcγR dependent (Figure S5C,D).”

To:

"TNP-liposome binding to cells incubated with anti-TNP-IgG2c was decreased upon prior treatment of cells with Fc-blocking antibodies, indicating that the binding was FcγR dependent (Figure S5C,D)."

11. Page 8
General comment on liposomes to engage Siglecs
Did you test if the liposomes expressing ligands for an specific Siglec bound U937 cells over expressing another Siglec? Could you explain further how the specific ligands were selected?

Reply: This is an important point brought up by the reviewer. The selectivity of these ligands is a major strength of our work and significant effort has gone into developing these ligands. The ligands for Siglec-3 and -9 were previously developed (PMID: 24921038, 23038623) and already shown to be selective for their target. Data in our manuscript clearly demonstrate the specificity of these ligands for their target. Specifically, the binding studies documents in Fig. 1f,h,j of our manuscript demonstrated that binding was completely abrogated in cells lacking that Siglec. The functional studies also confirm their specificity because the ligands produce no effects when the respective Siglec is not expressed.

12. Page 9
Lines 14-21
"Specifically, we tested activation of FcRs in cells with and without an individual Siglec. An internally-controlled assay was developed in which one cell type was labelled with carboxyfluorescein succinimidyl ester (CFSE) dye and mixed with another unstained cell type (Figure 2A).
I found this experimental design very difficult to follow.

Reply: The reason why we used this approach is that it enables two cell types to be analyzed at the same time under the exact same conditions. We have made the following motivation to our revised our initial manuscript from:

"An internally-controlled assay was developed in which one cell type was labeled with carboxyfluorescein succinimidyl ester (CFSE) dye and mixed with another unstained cell type (Figure 2A)."

To:

"An internally-controlled assay was developed in which one set of cells was labeled with carboxyfluorescein succinimidyl ester (CFSE) dye. These stained cells could then be mixed with a different set of unstained cells to examine the activation of two cell types under the exact same conditions, at the same time (Figure 2A)."

13. Lines 23-27
"Trinitrophenyl-bovine serum albumin (TNA-BSA) was used to stimulate the cell mixture and calcium flux was monitored by flow cytometry. The results demonstrated that the presence of an individual Siglec does not impact the cellular activation
why didn't you use TNP-bearing liposomes?

Reply: This is a good point brought up by the reviewer. There is a very good reason we initially chose TNP-BSA. In the manuscript, we didn't want to go too deep into the explanation so as to get distracted from the main take-home message. However, we now see that it could be confusing on why we chose this platform. We will explain below and then describe some added language we have added to the text.

We initially used TNP-BSA to evaluate the intrinsic role of Siglecs in inhibiting FcγRs as TNP-BSA is less multivalent than the TNP liposome platform. It has been reported that highly multivalent antigens can exclude inhibitory receptors (PMID: 37653247), so we wanted to first rule out multivalency hiding intrinsic Siglec inhibition before progressing to using liposomes to force the receptors together. Nevertheless, we did end up repeating the results with TNP liposomes, which are also presented in the manuscript. To make it more clear why TNP-BSA was used, we have added the following language to our revised manuscript:

"Because highly multivalent antigens have been seen to exclude inhibitory receptors[54], TNP-BSA was initially chosen to assess intrinsic Siglec inhibition as it is less multivalent than liposomes."

14. Page 10
Line 54
"TNP and a Siglec Ligand"
Liposomes with only the Siglec ligand should be included as additional control?

Reply: We agree that this is a very important control. This control was in fact described in our original manuscript within the supplementary information (Figure S10A,B). In the text, the following is written to describe these results:

"Importantly, separate liposomes displaying only Sig-3L or TNP administered simultaneously did not result in decreased cellular activation as measured by calcium flux (Figure S10A,B)."

15. Page 14
Line 3
"AF647-PEG-DSPE"
please clarify this is to make liposomes fluorescent

Reply: Correct. To help clarify, in our revised manuscript we have changed the text from:

"These ligands were linked to PEGylated lipids and incorporated into liposomes containing a fluorophore (AlexaFluor 647; AF647)), as described previously,[50] to assess cell binding by flow cytometry."

To:

"These ligands were linked to PEGylated lipids and incorporated into liposomes containing a fluorophore (AlexaFluor 647; AF647)), as described previously,[52] to assess fluorescent liposome binding to cells by flow cytometry. "

16. Page 18
Line 7

"to Siglec-9 upon pervanadate-mediated stimulation"
explain what this means.

Reply: Pervanadate is a protein tyrosine phosphatase inhibitor commonly used to increase tyrosine phosphorylation in cells. To make it more clear on why it is used, we have modified our text from:

"There is also some evidence pointing towards both the ITIM and ITSM of Siglec-3 being phosphorylated after treatment with a protein tyrosine phosphatase inhibitor, pervanadate,[58]"

to

"There is also some evidence pointing towards both the ITIM and ITSM of Siglec-3 being phosphorylated after treatment with a protein tyrosine phosphatase inhibitor, pervanadate,[56] and our calcium flux data revealed a dominant role for Siglec-3 ITIM in inhibiting FcγRs, with a minor role for the ITSM."

17. Page 19
Lines 22-27
"We deleted Siglec-7 in the CMAS-/- cells then virally transduced them with empty lentiviral control or WT Siglec-7 (Figure S16B). CMAS-/- cells overexpressing Siglec-7 bound Sig-7L liposomes very well (Figure S16C). "
It might be pertinent to discuss if expression of CMAS is regulated in U937 cells

Reply: CMAS is the enzyme that transforms sialic acid into CMP-sialic acid, which is the donor substrate for sialylatransferases. To our knowledge this enzyme is maintained at fairly constant expression levels. As described in our responses to Review 2 above, while CMAS is essential at an organismal level, the viability of cell lines is not impaired when CMAS is deleted.

18. Page 22
Line 23
"Specifically, we loaded one cell type"
which cell type? Please specify.

Reply: We agree that the cell type could be specified here to make the cell-cell platform clearer. Thus, we changed the wording in our manuscript from:

"Specifically, we loaded one cell type with TNP-PEG-DSPE or TNP-PEG-DSPE and Sig-3L-PEG-DSPE to be our "stimulating cells" (Figure 6B). Cells prepared with or without Siglec-3 served as "responding cells". Stimulating cells can then be combined with responding cells to monitor cellular activation."

To:

"Specifically, we loaded U937 $CMAS^{-/-}$ cells with TNP-PEG-DSPE or TNP-PEG-DSPE and Sig-3L-PEG-DSPE to serve as our 'stimulating cells' (Figure 6B). U937 cells prepared for calcium flux, with or without Siglec-3, were designated as our 'responding cells'. Stimulating cells could then be combined with responding cells, and cellular activation could be monitored."

19. Lines 42-43
"CMAS-/- cells loaded with TNP-PEG-DSPE and/or Sig-9LPEG-DSPE (Figure 6D) also had Siglec-9-dependent inhibition of FcR"
Why do you need to use the CMAS-/- cells? If I remember correctly, Siglec 9-expressing U937 cells showed an effect when tested with the liposomes.

Reply: We decided to use U937 CMAS$^{-/-}$ cells as these cells have no cell surface sialic acid on them, which could potentially engage other Siglecs on responding cells. To make this more clear, in our manuscript we have changed this sentence from:

"U937 CMAS$^{-/-}$ cells were used as loading cells to ensure that endogenous Siglec ligands could not engage Siglec-3/9 or other Siglecs on responding cells."

To

"U937 CMAS$^{-/-}$ cells, deficient in cell surface sialic acid, were used as loading cells to ensure that endogenous Siglec ligands could not engage multiple Siglecs on responding cells."

20. Page 23
Line 36
"using K562 cells"
why these cells?

Reply: We chose K562 cells for three reasons: 1) they grow in suspension so there is no step in getting them off the plate by EDTA and/or trypsin, 2) they are of a similar lineage as U937 cells but are thought to be an earlier stage of hematopoiesis, therefore, they do not express an abundance of Siglecs that could bind the Siglec ligands in *cis*, and 3) stimulation of one cell type by a different cell type adds novelty and more closely resembles a natural immune cell-tumor cell interaction.

oc-2023-00969u.R2

Name: Peer Review Information for "Dissecting the ability of Siglecs to antagonize Fcg receptors"

Second Round of Reviewer Comments

Reviewer: 2

Comments to the Author

All my questions have been addressed by the authors.

Author's Response to Peer Review Comments:

TOC: Please move to the last page of the manuscript, beneath the References.

-done

SYNOPSIS MISSING: The synopsis should be no more than 200 characters (including spaces) and should reasonably correlate with the TOC graphic. The synopsis is intended to explain the importance of the article to a broader readership across the sciences. Please place your synopsis in the manuscript file after the TOC graphic, and label it as “Synopsis.”

-done

SI PARAGRAPH: If the manuscript is accompanied by any supporting information for publication, a brief description of the supplementary material is required in the manuscript. The appropriate format is: Supporting Information. Brief statement in non-sentence format listing the contents of the material supplied as Supporting Information.

-done

ENERAL REF FORMATTING: Periodical references should contain authors’ surnames followed by initials, article title, journal abbreviation, year, volume number, and page range. Refs with more than 10 authors should list the first 10 and then be followed by “et al.”

-fixed

Web sources must include access date.

-N/A

SI PG#S: The supporting information pages must be numbered consecutively, starting with page S1.

-done

HIGHLIGHTING: Please remove the highlighting in the text of both the Manuscript and Supporting Information files, and upload “clean” copies for publication. You may upload annotated files separately as “Supporting Information for Review Only” files.

-done
